# Supplementary material for: AAV-delivered muscone-induced transgene system for treating chronic diseases in mice via inhalation
Source: Nat Commun. 2024 Feb 6;15:1122. doi: 10.1038/s41467-024-45383-z (PMC10847102; doi:10.1038/s41467-024-45383-z)
Supplement: Supplementary file 1 — Supplementary Information [file 41467_2024_45383_MOESM1_ESM.pdf]

Supplementary Information

AAV-delivered muscone-induced transgene system for treating chronic diseases in mice via inhalation

Xin Wu[1,2,7], Yuanhuan Yu[1,7], Meiyan Wang[1,3,7], Di Dai[1], Jianli Yin[1,3], Wenjing Liu[1], Deqiang Kong[1], Shasha Tang[4], Meiyao Meng[1], Tian Gao[1], Yuanjin Zhang[1], Yang Zhou[1,5], Ningzi Guan[1], Shangang Zhao[6], and Haifeng Ye[1,3,5]*

[1]Shanghai Frontiers Science Center of Genome Editing and Cell Therapy, Biomedical Synthetic Biology Research Center, Shanghai Key Laboratory of Regulatory Biology, Institute of Biomedical Sciences and School of Life Sciences, East China Normal University, Dongchuan Road 500, Shanghai 200241, China.

[2]Institute of Medical Technology, Shanxi Medical University, Taiyuan, Shanxi Province 030001, China.

[3]Chongqing Key Laboratory of Precision Optics, Chongqing Institute of East China Normal University, Chongqing 401120, China.

[4]Department of Breast Surgery, Tongji Hospital, School of Medicine, Tongji University, Xincun Road 389, Shanghai 200065, China.

[5]Wuhu Hospital, Health Science Center, East China Normal University, Middle Jiuhua Road 263, Wuhu, Anhui, China.

[6]Sam and Ann Barshop Institute for Longevity and Aging Studies, Division of Endocrinology, Department of Medicine, University of Texas Health Science Center at San Antonio, San Antonio, TX, 78229, USA.

[7]These authors contributed equally.

*To whom correspondence should be addressed: E-mail: hfye@bio.ecnu.edu.cn

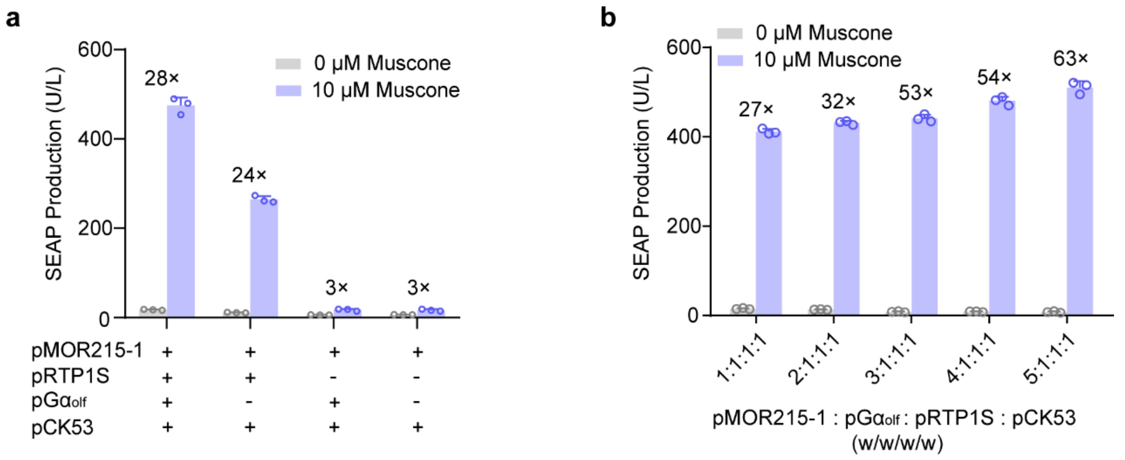

Supplementary Fig. 1 Optimization of a muscone-induced transgene system (MUSE) in HEK-293T cells. (**a**) Optimization of the different combinations of plasmids encoding MUSE. In total, 6×10^4 HEK-293T cells were co-transfected with different combinatorial configurations of the plasmids encoding the muscone-responsive receptor expression vector pMOR215-1 (P_{SV40}-MOR215-1-pA), the cAMP-responsive promoter (P_{CRE}) driven reporter expression vector pCK53 (P_{CRE}-SEAP-pA), a truncated version of the receptor-transporting protein vector pRTP1S (P_{SV40}-RTP1S-pA), and an olfactory neuron-specific G protein alpha subunit vector pGα_{olf} (P_{SV40}-Gα_{olf}-pA). Transfected cells were cultivated in a culture medium with or without 10 μM muscone for 48 h, and SEAP production in the culture supernatant was profiled. (**b**) Optimization of the different ratios of plasmids encoding pMOR215-1, pRTP1S, pGα_{olf}, and pCK53. HEK-293T cells were co-transfected with the plasmids encoding pMOR215-1, pRTP1S, pGα_{olf}, and pCK53 at different ratios (w/w/w/w) and cultivated in culture medium with or without 10 μM muscone for 48 hours. SEAP production in the culture supernatant was profiled. Data are presented as means ± SD; *n* = 3 biologically independent samples. All plasmids are described in Supplementary Tables 1 and 3. Source data are provided as a Source Data file.

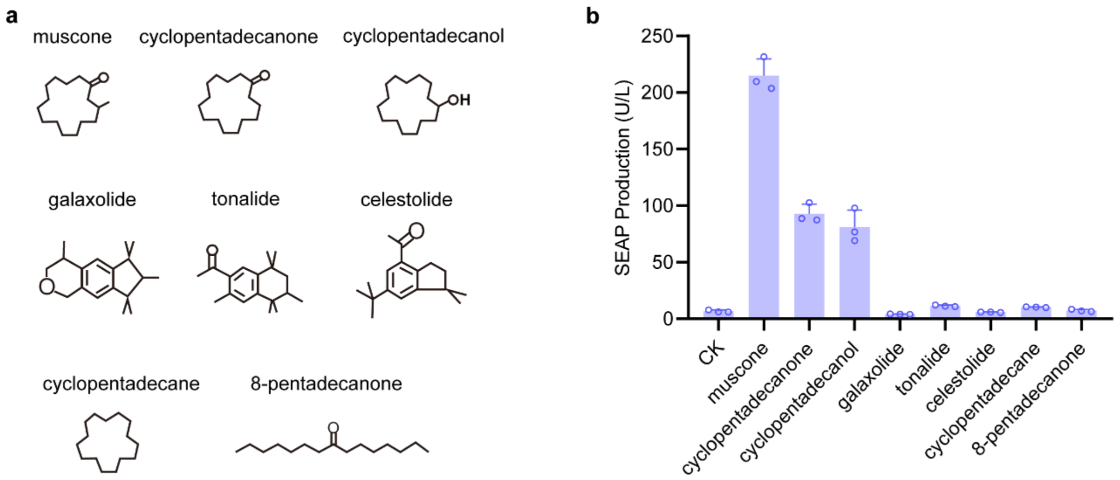

Supplementary Fig. 2 Cross-reactivity of AAV$_{MUSE}$ to musk-related chemicals. pWX126/pWX127/pWX123-transgenic HEK-293T cells were incubated with muscone [$C_{16}H_{30}O$], cyclopentadecanone [$C_{15}H_{28}O$], cyclopentadecanol [$C_{15}H_{30}O$], galaxolide [$C_{18}H_{26}O$], tonalide [$C_{18}H_{26}O$], celestolide [$C_{17}H_{24}O$], cyclopentadecane [$C_{15}H_{30}$] and 8-pentadecanone [C_{15}H30O]) at 10 µM. SEAP expression was profiled in the cell culture supernatant after 24 hours. Data are presented as means ± SD; n = 3 biologically independent samples. All plasmids are described in Supplementary Tables 1 and 3. Source data are provided as a Source Data file.

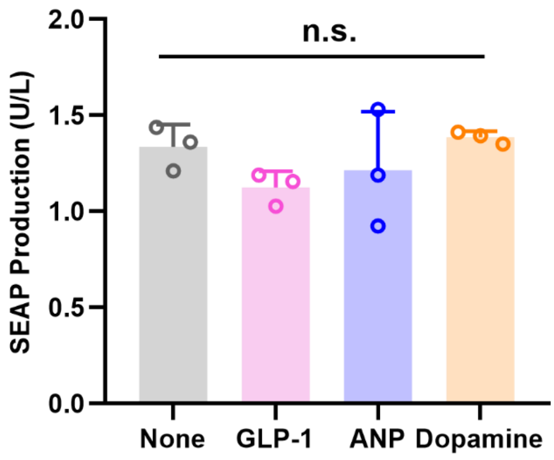

Supplementary Fig. 3 Insensitivity of AAV$_{MUSE}$ to endogenous cAMP-agonists. HEK-293T cells were co-transfected with pWX126, pWX127, and pWX158 and incubated with multiple cAMP-agonists at their physiological concentrations. SEAP levels in the culture supernatants were quantified at 24 hours. Data are presented as means ± SD; n = 3 biologically independent samples. P values were obtained using one-way ANOVA. One-way ANOVA, followed by Dunnett's post hoc test, was used to compare multiple groups. n.s., not significant. All plasmids are described in Supplementary Tables 1 and 3. Source data are provided as a Source Data file.

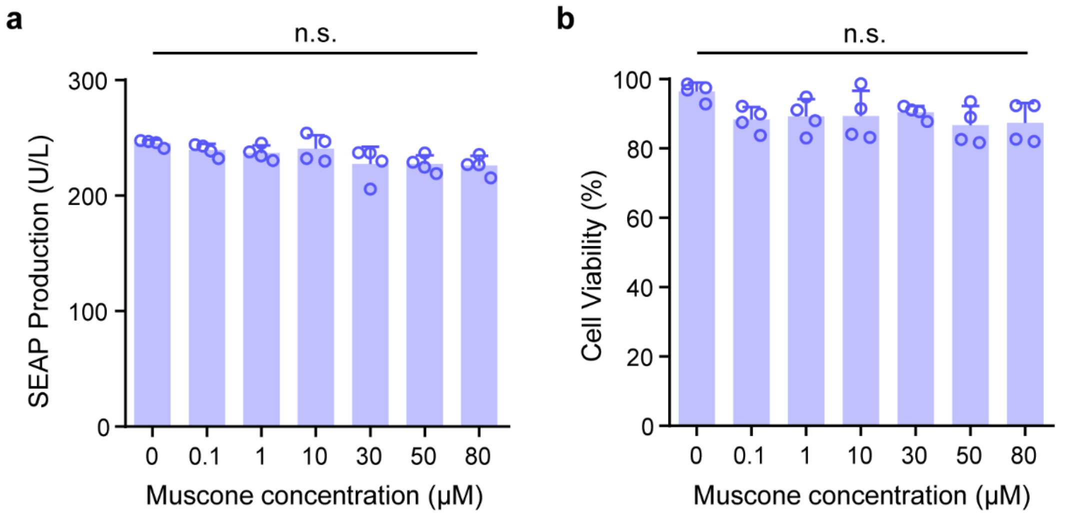

Supplementary Fig. 4 Assessing the cytotoxicity of muscone to cells. (**a**) Protein-based metabolic integrity assay of HEK-293T cells exposed to muscone. In total, 5×10^4 HEK-293T cells were transfected with 0.2 μg pSEAP2-control (P_{SV40}-SEAP-pA) and cultivated in a medium containing various concentrations of muscone (0-80 μM) for 72 h; SEAP in the culture supernatant was profiled. (**b**) Cell viability of HEK-293T cells after exposure to various concentrations of muscone. A total of 5×10^4 HEK-293T cells were cultivated in a medium containing the indicated muscone concentrations (0-80 μM) for 72 h, and cell viability was evaluated using a CCK-8 Kit. Data are presented as means ± SD; n = 4 biologically independent samples. P values were obtained using one-way ANOVA. One-way ANOVA, followed by Dunnett's post hoc test, was used to compare multiple groups. n.s., not significant. All plasmids are described in Supplementary Tables 1 and 3. Source data are provided as a Source Data file.

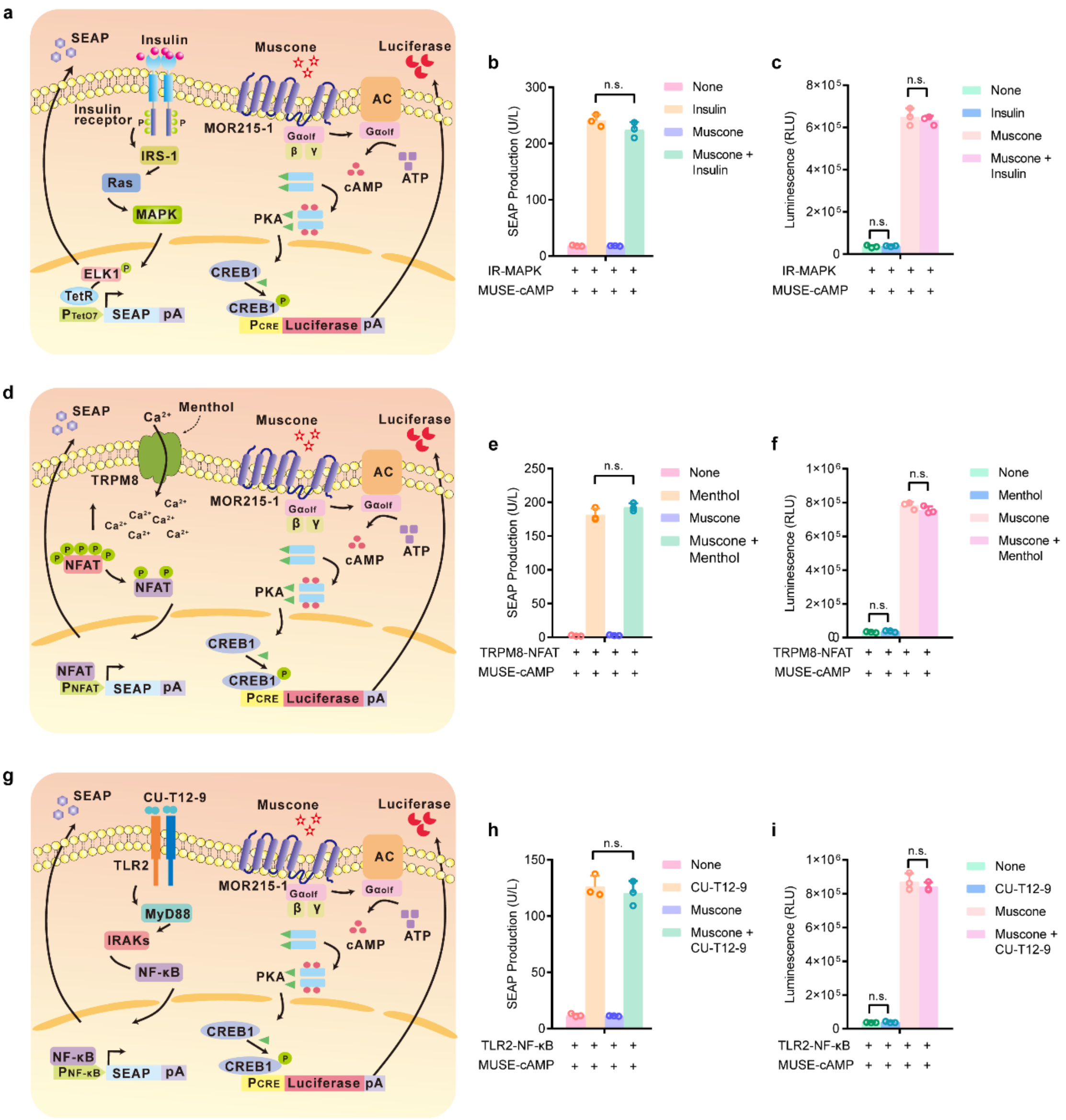

Supplementary Fig. 5 Crosstalk analysis between the MUSE-mediated cAMP signaling pathway and three other signaling pathways. **(a)** Schematic representations of the MUSE-mediated cAMP signaling pathway and the insulin receptor (IR)-mediated MAPK signaling pathways. **(b, c)** Crosstalk analysis between the MUSE mediated-cAMP signaling pathway and the IR-mediated MAPK signaling pathway. HEK-293T cells were co-transfected with plasmids expressing luciferase under the control of the MUSE-mediated cAMP signaling pathway and plasmids encoding secreted alkaline phosphatase (SEAP) expression under the control of the IR-mediated MAPK signaling pathway. Transfected cells were cultured in the presence or absence of insulin (20 ng/mL) with or without muscone (10 μM). **(d)** Schematic representations of the MUSE-mediated cAMP signaling pathway and the transient

receptor potential (TRP) melastatin 8 (TRPM8)-mediated NFAT signaling pathway. (**e, f**) Crosstalk analysis between the MUSE-mediated cAMP signaling pathway and the TRPM8-mediated NFAT signaling pathway. HEK-293T cells were co-transfected with plasmids expressing luciferase under the control of the MUSE-mediated cAMP signaling pathway and plasmids encoding SEAP expression under the control of the TRPM8-mediated NFAT signaling pathway. Transfected cells were cultured in the presence or absence of menthol (50 μM) with or without muscone (10 μM). (**g**) Schematic representations of the MUSE-mediated cAMP signaling pathway and the toll-like receptor 2 (TLR2)-mediated NF-κB signaling pathway. (**h, i**) Crosstalk analysis between the MUSE-mediated cAMP signaling pathway and the TLR2-mediated NF-κB signaling pathway. HEK-293T cells were co-transfected with plasmids expressing luciferase under the control of the MUSE-mediated cAMP signaling pathway and plasmids encoding SEAP expression under the control of the plasmids encoding TLR2-mediated NF-κB signaling pathway. Transfected cells were cultured in the presence or absence of CU-T12-9 agonists (50 μM) with or without muscone (10 μM). SEAP and luciferase expression were profiled 24 hours after induction. Data are presented as means ± SD; n = 3 biologically independent samples. P values were obtained from two-tailed unpaired t-tests. n.s., not significant. All plasmids are described in Supplementary Tables 1 and 3. Source data are provided as a Source Data file.

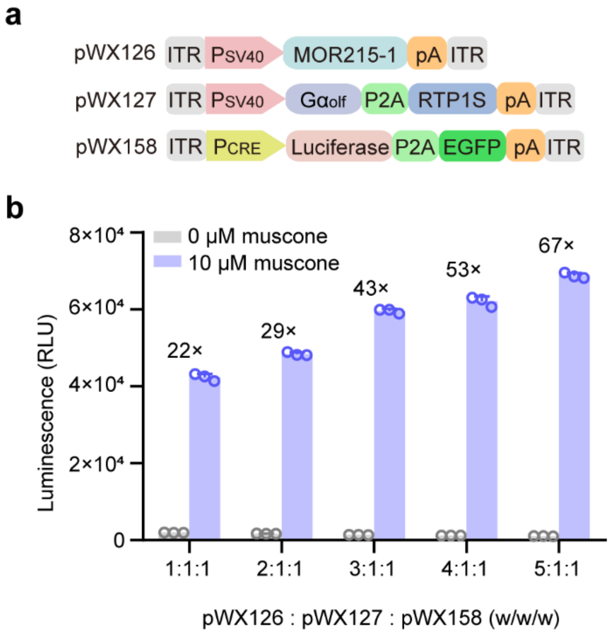

Supplementary Fig. 6 Optimization of the different ratios of plasmids encoding AAV$_{MUSE}$ in HEK-293T cells. (**a**) Schematic representing genetic configurations of the plasmid vector encoding AAV$_{MUSE}$. (**b**) Optimization of the different ratios of plasmids encoding AAV$_{MUSE}$. HEK-293T cells were co-transfected with the muscone-responsive receptor expression vector pWX126 (ITR-P$_{SV40}$-pMOR215-1-pA-ITR), a concatenated Gα_{olf} and RTP1S vector pWX127 (ITR-P$_{SV40}$-Gα_{olf}-P2A-RTP1S-pA-ITR), and P$_{CRE}$-responsive reporter protein expression vector pWX158 (ITR-P$_{CRE}$-Luciferase-P2A-EGFP-pA-ITR) at different ratios (w/w/w) and cultivated in culture medium with or without 10 μM muscone for 48 h, and luciferase levels were profiled. Data in b are presented as means ± SD; n = 3 biologically independent samples. All plasmids are described in Supplementary Tables 1 and 3. Source data are provided as a Source Data file.

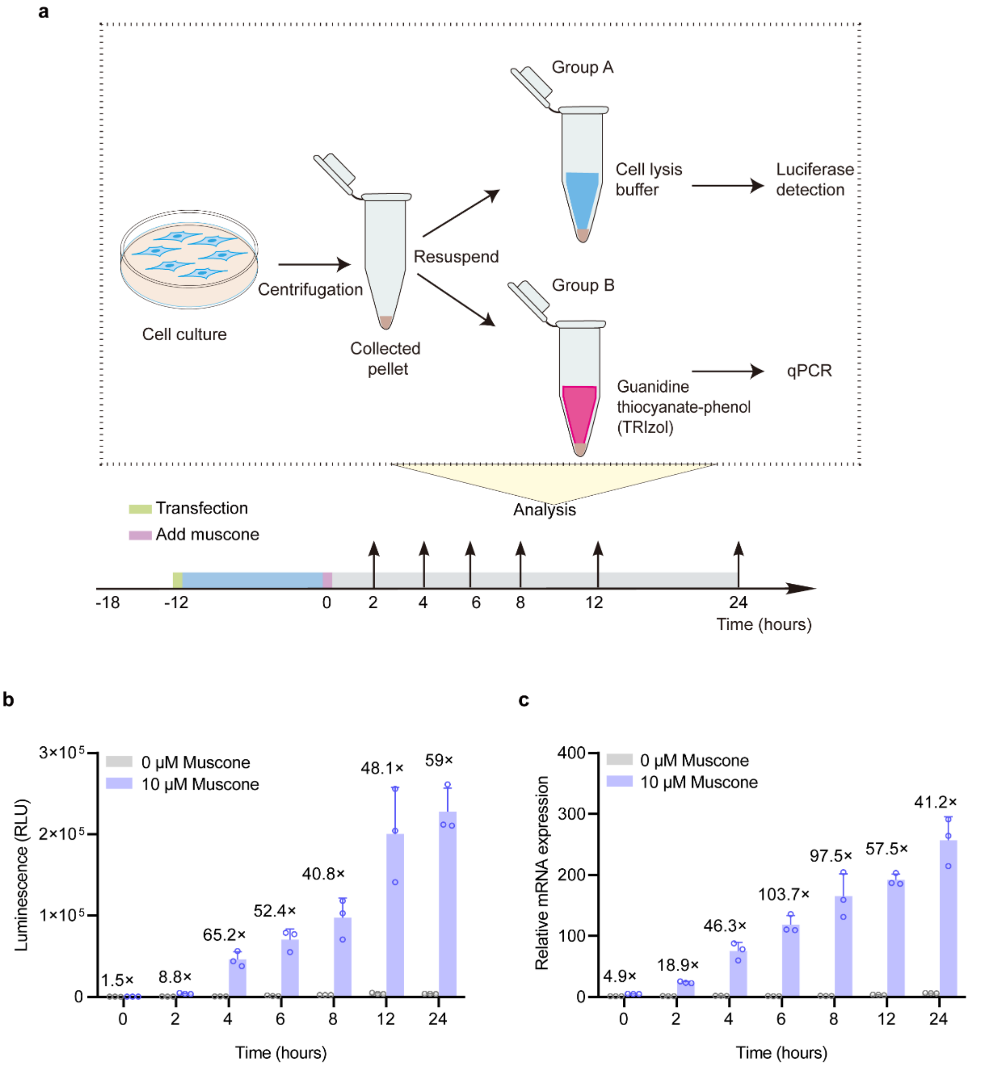

Supplementary Fig. 7 The kinetic of AAV$_{\text{MUSE}}$-mediated luciferase expression *in vitro*. (**a**) Schematic for the schedule and experimental procedure for the muscone-controlled gene expression. HEK-293T cells (6×10^4) transfected with the AAV$_{\text{MUSE}}$ system (pWX126, pWX127, pWX158) were cultivated for the indicated periods (0 to 24 hours) in the presence of 0 or 10 µM muscone. Then cells were collected immediately at the indicated time points (X-axis, 0 to 24 hours) and divided into two groups; one (group A) was used to measure luciferase activity (**b**), and the other one (group B) was collected to extract RNA for qPCR analysis of the luciferase reporter (**c**). Data are presented as means ± SD; $n = 3$ biologically independent samples. All plasmids are described in Supplementary Tables 1 and 3. The sequences of primer pairs used in the study are listed in Supplementary Table 2. Source data are provided as a Source Data file.

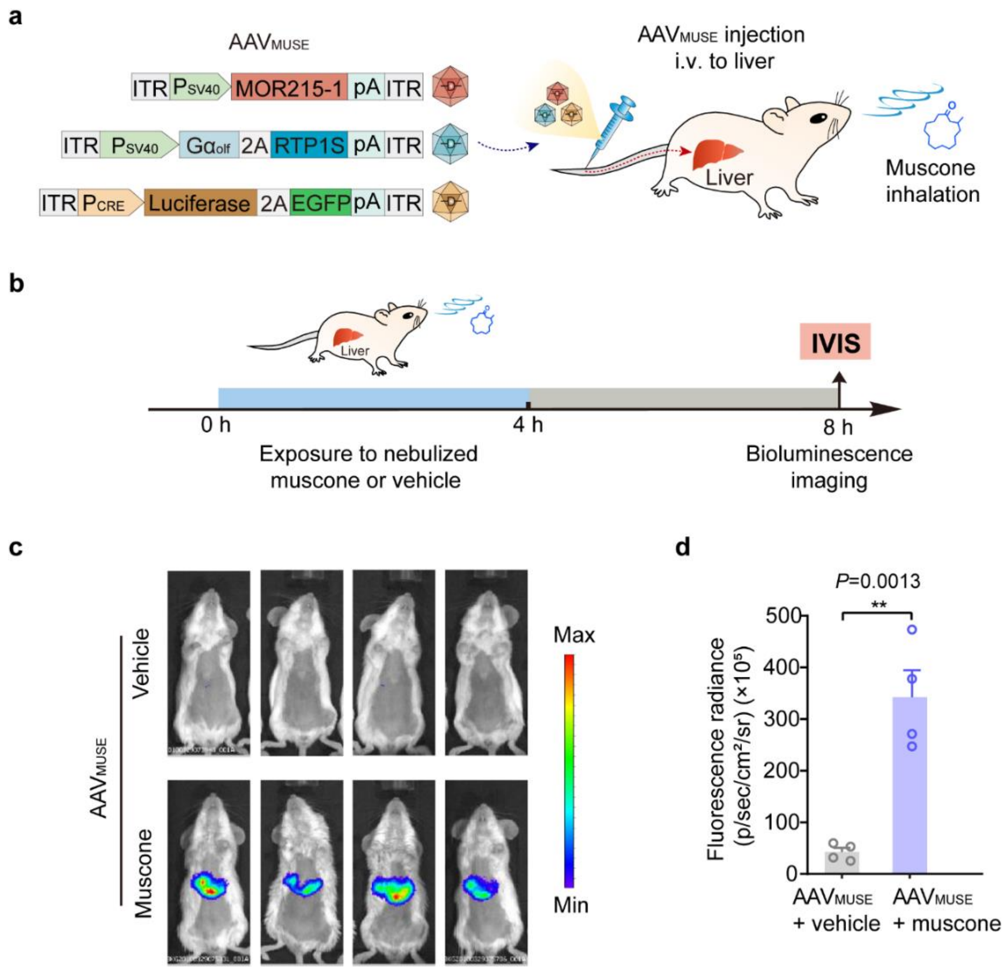

Supplementary Fig. 8 AAV$_{MUSE}$-mediated luciferase expression in mouse livers upon exposure to nebulized muscone. (**a**) Schematic representation of the experimental procedure for AAV$_{MUSE}$-mediated luciferase expression in mice. Female BALB/c mice (8-week-old) were transduced with AAV2/9 vectors carrying the AAV$_{MUSE}$ components via tail vein injection. At 2 weeks after AAV injection, AAV$_{MUSE}$-transduced mice were exposed to nebulized muscone or vehicle (a mixture of castor oil and ddH_2O). (**b**) Schematic representation of the time schedule for AAV$_{MUSE}$-mediated luciferase expression in mice. AAV$_{MUSE}$-transduced mice were exposed to a nebulized muscone or vehicle for 4 hours. (**c**) The bioluminescence signal was quantified 4 hours after exposure to a nebulized muscone using an *in vivo* imaging system. (**d**) Bioluminescence measurements of the muscone-induced luciferase expression mediated by AAV$_{MUSE}$ based on bioluminescence imaging in c. Data in d are presented as means ± SEM (n = 4 mice). P values were obtained from two-tailed

unpaired *t*-tests. ***P* < 0.01. All plasmids are described in Supplementary Tables 1 and 3. Source data are provided as a Source Data file.

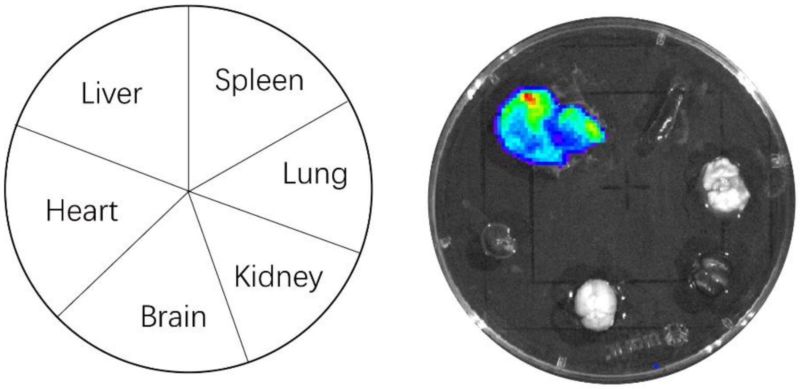

Supplementary Fig. 9 Bioluminescence imaging of mouse tissues. Female BALB/c mice (8-week-old) were transduced with AAV_{MUSE} containing the muscone-responsive vector AAV2/9-pWX126 (3×10^{11} vg), the concatenated $G\alpha_{olf}$ and RTP1S expression vector AAV2/9-pWX127 (1.5×10^{11} vg), and the inducible reporter vector AAV2/9-pWX158 (ITR-P_{CRE}-luciferase-P2A-EGFP-pA-ITR, 1.5×10^{11} vg) via tail vein injection. Two weeks after the AAV injection, AAV_{MUSE}-transduced mice were exposed to nebulized muscone for 4 hours using an ultrasonic nebulizer. Then, the mice were euthanized, and the liver, brain, heart, spleen, lung, and kidney were dissected for bioluminescence imaging. Representative bioluminescence images of mouse tissues from the AAV_{MUSE}-transduced mice were shown.

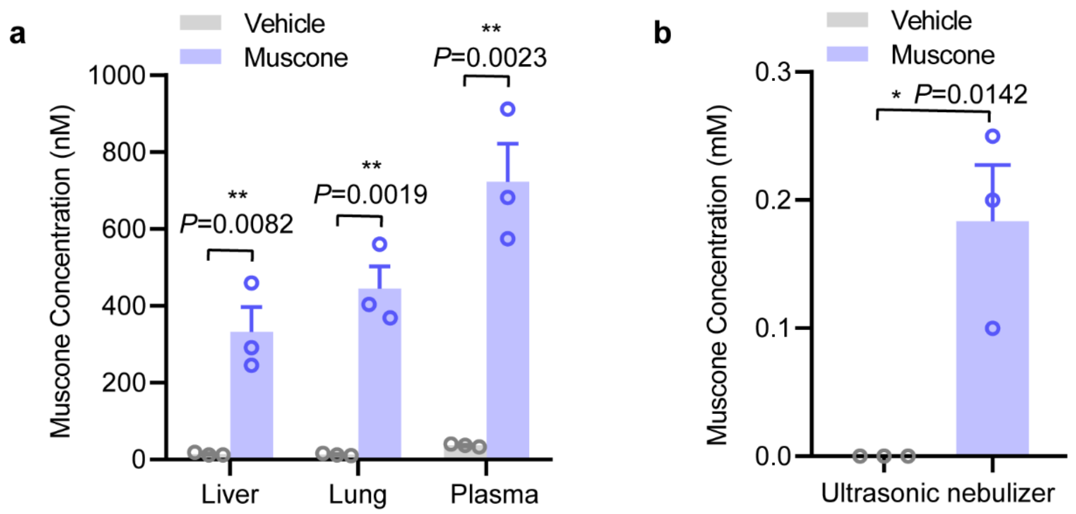

Supplementary Fig. 10 Quantification of the muscone concentration by GC-MS. (**a**) Muscone concentration was quantified in the liver, lung, and plasma of mice exposed to nebulized muscone. (**b**) Measurement of muscone in the headspace of ultrasonic nebulizer by GC-MS. Data are presented as means ± SEM (n = 3 mice). P values were obtained from two-tailed unpaired t-tests. *P < 0.05, **P < 0.01. Source data are provided as a Source Data file.

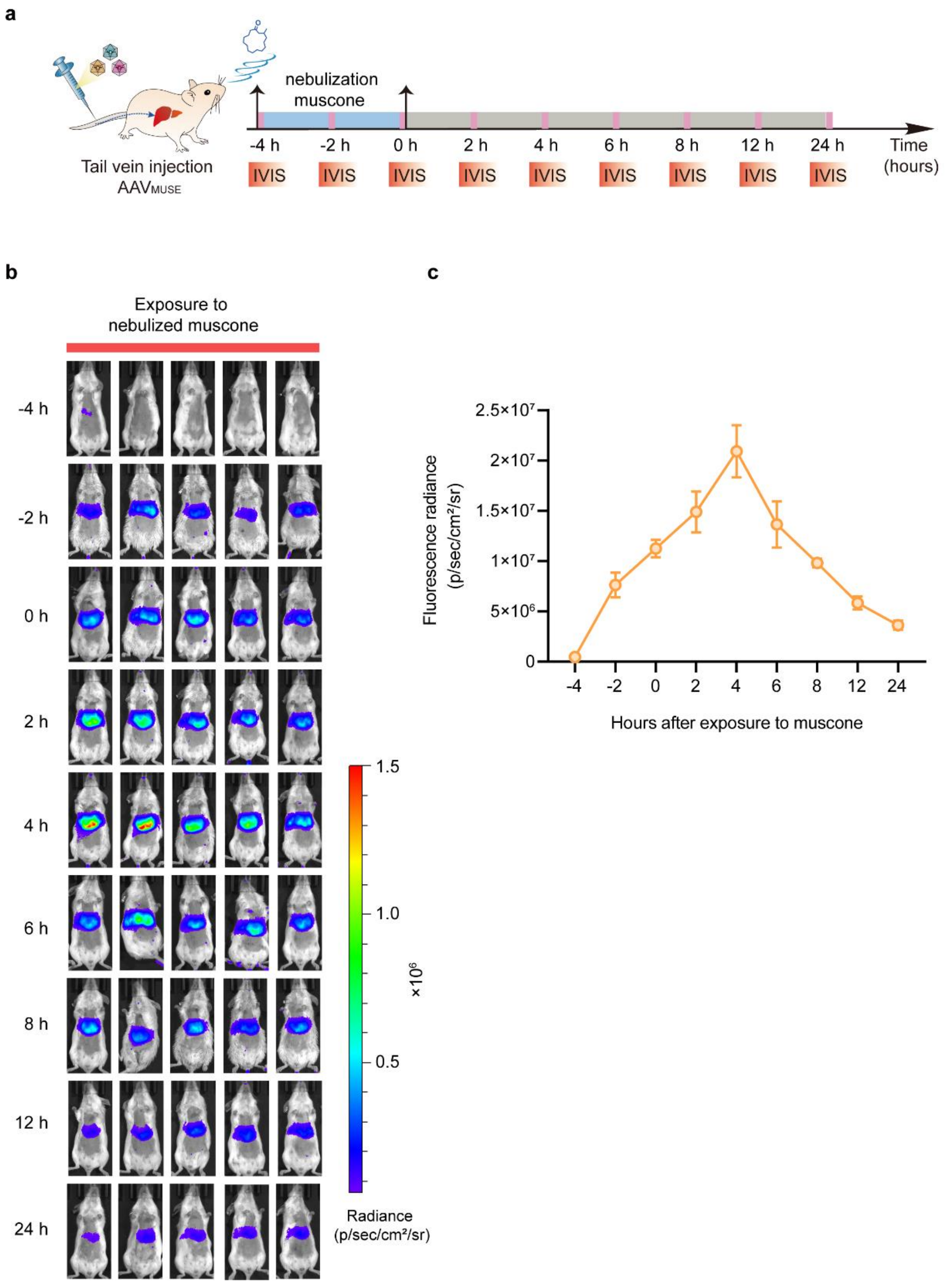

Supplementary Fig. 11 Kinetics of AAV$_{\mathrm{MUSE}}$-mediated luciferase expression *in vivo*. (a) Schematic representation of the experimental procedure and the time schedule for AAV-2/9 delivery of AAV$_{\mathrm{MUSE}}$-mediated transgene expression in mice. The time range from -4 to 0 h represents the duration of nebulized muscone, with 0 h indicating the cessation of nebulization. (**b**) Time-dependent AAV$_{\mathrm{MUSE}}$-mediated transgene expression kinetics in mice. BALB/c mice were injected with the AAV$_{\mathrm{MUSE}}$ containing

the muscone-responsive vector AAV2/9-pWX126 (3×10^{11} vg), the concatenated $G\alpha_{olf}$ and RTP1S expression vector AAV2/9-pWX127 (1.5×10^{11} vg), and the inducible reporter vector AAV2/9-pWX158 (ITR-P_{CRE}-luciferase-P2A-EGFP-pA-ITR, 1.5×10^{11} vg) via tail vein injection. Two weeks after AAV injection, AAV_{MUSE}-transduced mice were exposed to nebulized muscone using an ultrasonic nebulizer, and bioluminescence imaging was monitored at the indicated periods (-4 hours to 24 hours) using an *in vivo* imaging system. (**c**) Bioluminescence measurements of the luciferase expression based on bioluminescence imaging in b. Data are presented as means ± SEM (n = 5 mice). Each dot represents an individual mouse. Source data are provided as a Source Data file.

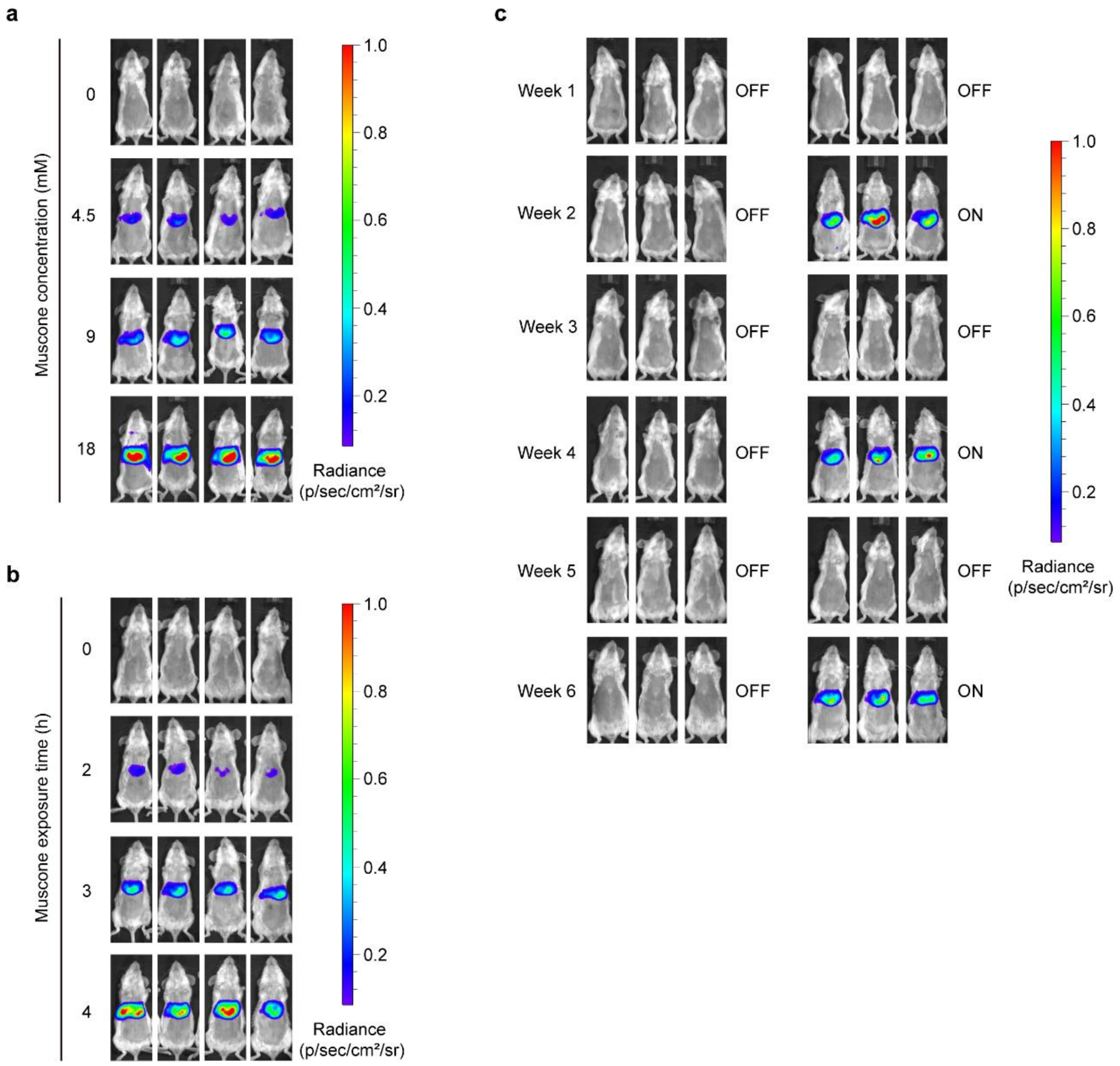

Supplementary Fig. 12 Detailed images of the bioluminescence signal resulting from AAV$_{MUSE}$-mediated luciferase expression for each mouse in Figure 3.

(**a**) Detailed images of the bioluminescence signal in Figure 3b. (**b**) Detailed images of the bioluminescence signal in Figure 3c. (**c**) Detailed images of the bioluminescence signal in Figure 3d.

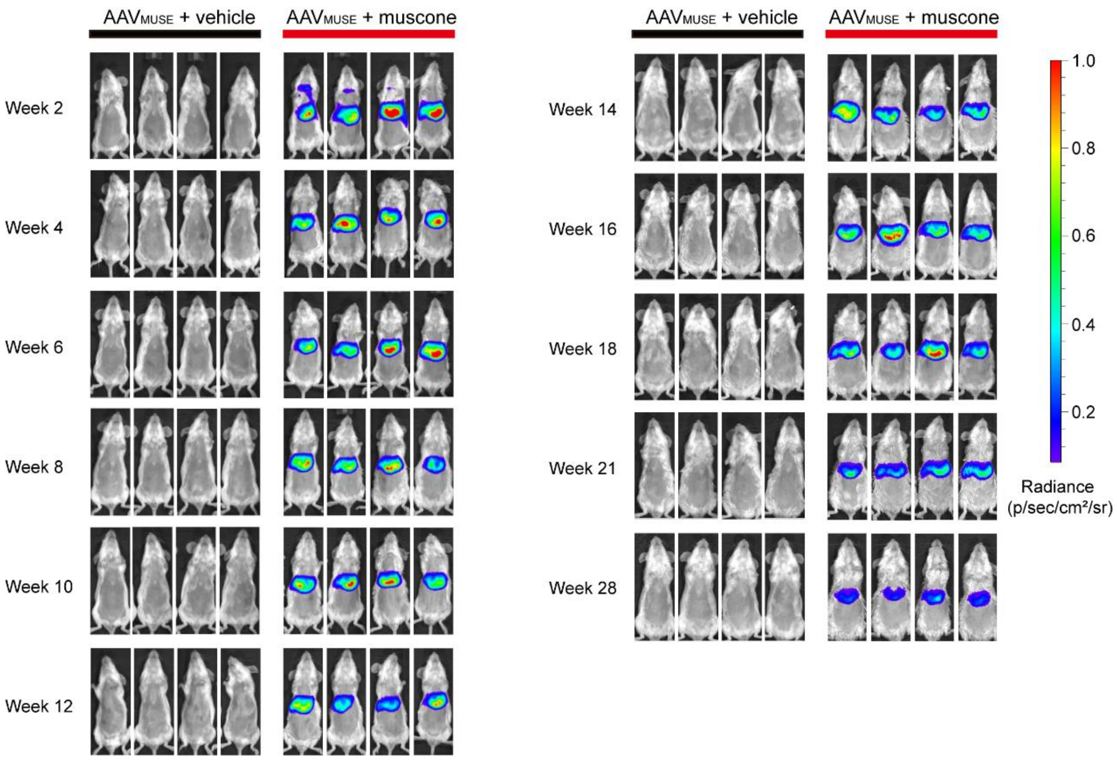

Supplementary Fig. 13 Long-term study of AAV$_{MUSE}$-mediated luciferase expression in mice (related to Figure 3f). BALB/c mice were transduced with AAV$_{MUSE}$ containing the muscone-responsive receptor expression vector AAV2/9-pWX126 (3×10^{11} vg), the concatenated Gα_{olf} and RTP1S expression vector AAV2/9-pWX127 (1.5×10^{11} vg), and inducible reporter expression vector AAV2/9-pWX158 (ITR-P$_{CRE}$-luciferase-P2A-EGFP-pA-ITR, 1.5×10^{11} vg) via tail vein injection. Two weeks after AAV injection, AAV$_{MUSE}$-transduced mice were exposed to a nebulized muscone or vehicle for 4 hours once every two weeks. Bioluminescence imaging was performed at the indicated time points. $n = 4$ mice.

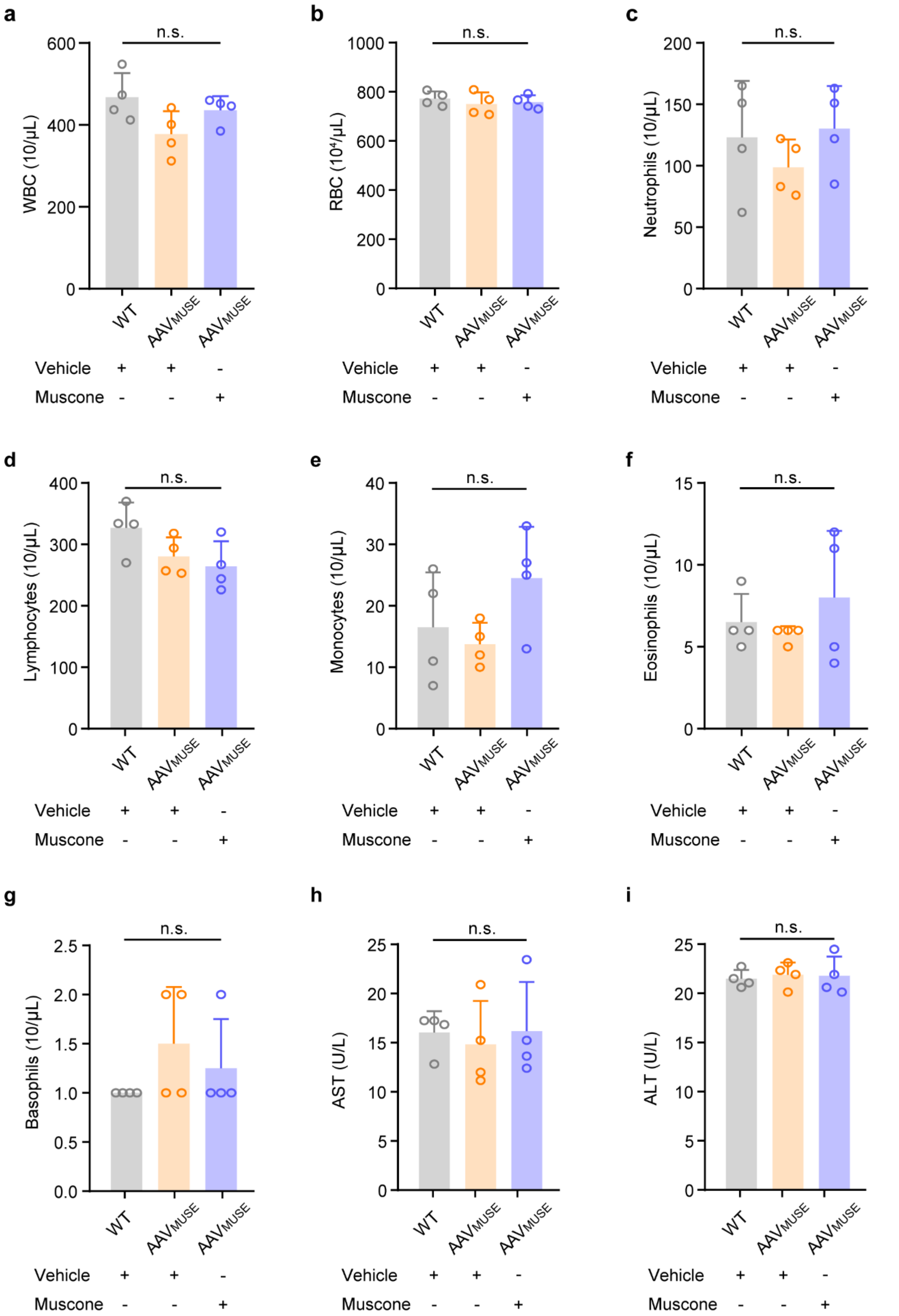

Supplementary Fig. 14 Routine blood test and serum biochemistry analysis on AAV$_{MUSE}$-transduced mice exposed to muscone or vehicle, as well as on untreated wild-type BALB/c mice. (**a-i**) Female BALB/c mice (8-week-old) were transduced with AAV2/9 vectors carrying the AAV$_{MUSE}$ components. Two weeks after AAV

injection, AAV_{MUSE}-transduced mice were exposed to a nebulized muscone or vehicle for 4 hours once every two weeks. Control wild-type BALB/c mice did not receive either AAV_{MUSE} or muscone. Twenty-eight weeks after AAV_{MUSE} injection, mouse blood was collected for blood biochemical analysis including counts of (**a**) total white blood cells (WBC), (**b**) red blood cells (RBC), (**c**) neutrophils, (**d**) lymphocytes, (**e**) monocytes, (**f**) eosinophils, (**g**) basophils, and for hepatic function analysis including (**h**) aspartate aminotransferase (AST) and (**i**) alanine aminotransferase (ALT). Data are expressed as means ± SEM (n = 4 mice). P values were obtained using one-way ANOVA. One-way ANOVA, followed by Dunnett's post hoc test, was used to compare multiple groups. n.s., not significant. Source data are provided as a Source Data file.

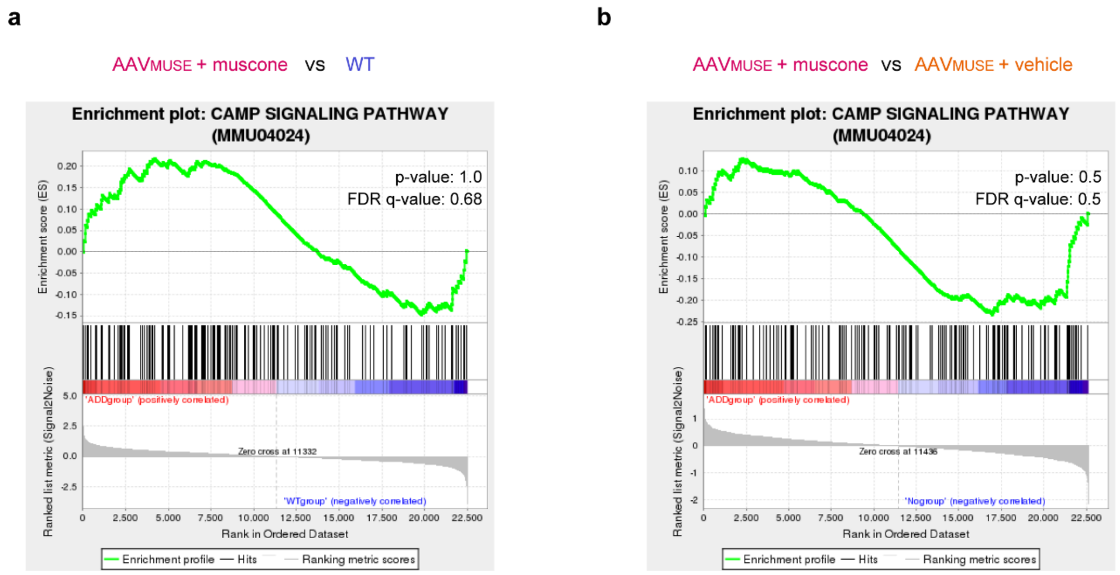

Supplementary Fig. 15 Analysis of potential signaling pathways influenced by cAMP induced by the MUSE system through Gene Set Enrichment Analysis (GSEA). (**a**) GSEA for cAMP pathway in the livers of AAV_{MUSE}-transduced mice exposed to nebulized muscone versus control wild-type mice. (**b**) GSEA for cAMP pathway in the livers of AAV_{MUSE}-transduced mice exposed to nebulized muscone versus AAV_{MUSE}-transduced mice exposed to vehicle. Tissue samples were collected for GSEA testing analysis after a total muscone induction period of 20 weeks, with 4 hours each week. FDR: false discovery rate. $n = 5$ mice.

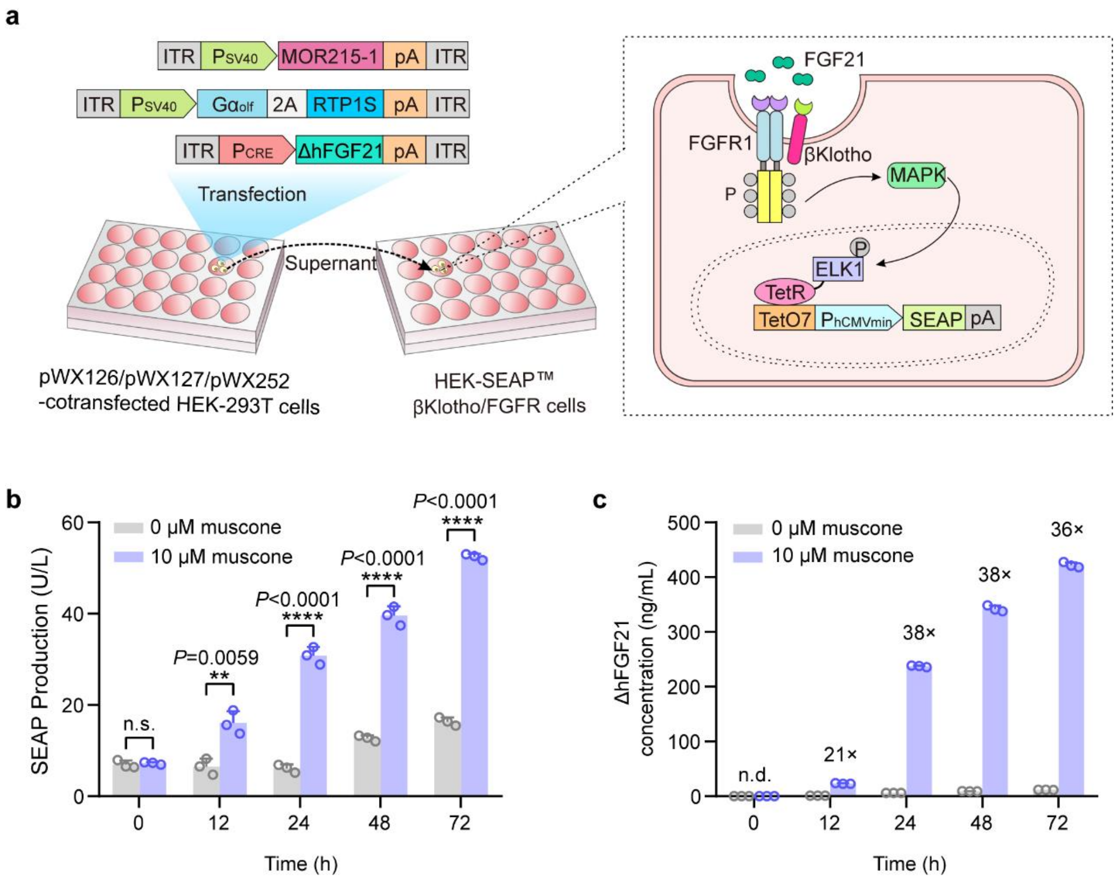

Supplementary Fig. 16 Validation of the biological activity of ΔhFGF21 and characterization of AAV$_{\mathrm{MUSE}}$-mediated ΔhFGF21 expression kinetics. (**a**) Experimental procedure for functional assessment of ΔhFGF21 activity through an engineered HEK-SEAP™ βKlotho/FGFR cell line. Engineered cells were co-transfected with pβKlotho (P_{CMV}-βKlotho-pA; 100 ng), pTetR-ELK1 (P_{CMV}-TetR-ELK1-pA; 100 ng), and pMF111 ($P_{hCMV*-1}$-SEAP-pA; $P_{hCMV*-1}$, O_{TetO7}-$P_{hCMVmin}$; 100 ng). When ΔhFGF21 bound to FGFR1 and its coreceptor β-Klotho, the MAPK signaling pathway was activated to phosphorylate TetR-ELK1 (a synthetic hybrid transcription factor), leading to $P_{hCMV*-1}$-driven SEAP reporter expression in the engineered cells. (**b**) Time-dependent expression of SEAP in the engineered cells. HEK-293T cells were co-transfected with pWX126 (ITR-P_{SV40}-MOR215-1-pA-ITR; 250 ng), pWX127 (ITR-P_{SV40}-Gα_{olf}-P2A-RTP1S-pA-ITR; 125 ng), and pWX252 (ITR-P_{CRE}-ΔhFGF21-pA-ITR; 125 ng) at a ratio of 2:1:1 (w/w/w) and cultivated in culture medium with or without 10 μM muscone for 0 - 72 hours. Then, culture supernatant from pWX126/pWX127/pWX252-cotransfected HEK-293T cells was transferred to

HEK-SEAP™ βKlotho/FGFR cells, and SEAP production was profiled 24 hours after incubation. (**c**) Muscone exposure time-dependent AAV_{MUSE}-mediated ΔhFGF21 expression kinetics. pWX126/pWX127/pWX252-cotransfected HEK-293T cells were cultivated for the indicated periods (0 to 72 hours) with or without 10 μM muscone. ΔhFGF21 levels in the culture supernatant were quantified at the indicated time points (X-axis, 0 to 72 hours) using ELISA kits. Data in b and c are presented as means ± SD; $n = 3$ biologically independent samples. P values were obtained from two-tailed unpaired t-tests. ****$P < 0.0001$. n.s., not significant. n.d., not detectable. Descriptions of all plasmids, and detailed descriptions of the genetic constructs, are provided in Supplementary Tables 1 and 3. Source data are provided as a Source Data file.

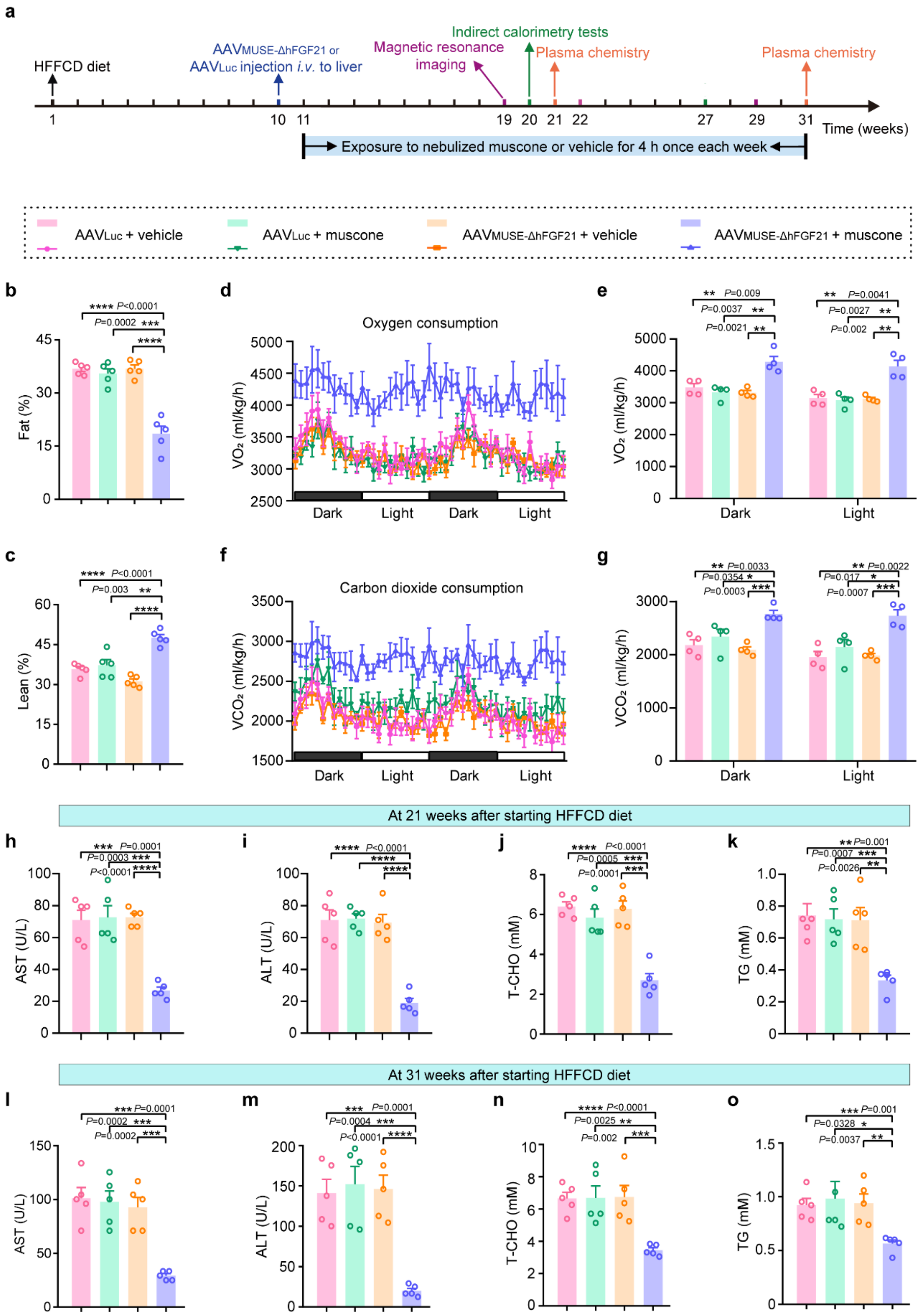

Supplementary Fig. 17 $AAV_{MUSE\text{-}\Delta hFGF21}$-mediated gene therapy in diet-induced non-alcoholic fatty liver disease (NAFLD) model mice. (**a**) Schematic representation of the experimental procedure and time schedule for AAV_{MUSE}-mediated ΔhFGF21 expression ($AAV_{MUSE\text{-}\Delta hFGF21}$) in NAFLD model mice. At 10 weeks after starting the

high-fat, high-fructose, high-cholesterol diet (HFFCD) (blue arrow), NAFLD model mice were transduced with $AAV_{MUSE\text{-}\Delta hFGF21}$ including the muscone-responsive receptor expression vector AAV2/9-pWX126 (1×10^{11} vg), the concatenated $G\alpha_{olf}$ and RTP1S expression vector AAV2/9-pWX127 (5×10^{10} vg), and the ΔhFGF21 inducible expression vector AAV2/9-pWX252 (ITR-P_{CRE}-ΔhFGF21-pA-ITR, 5×10^{10} vg), or transduced with control AAV_{Luc} (2×10^{11} vg). One week after AAV injection, the transduced NAFLD model mice were exposed to nebulized muscone for 4 hours once each week until week 31 or were exposed to a vehicle (a mixture of castor oil and ddH_2O). The examined controls included NAFLD model mice transduced with $AAV_{MUSE\text{-}\Delta hFGF21}$ and exposed to a vehicle, and NAFLD model mice transduced with AAV_{Luc} with or without exposure to muscone. (**b**) The fat mass and (**c**) lean mass were determined by magnetic resonance imaging at 19 weeks after starting the HFFCD diet (purple arrow). (**d-g**) Indirect calorimetry was performed 20 weeks after starting the HFFCD diet (green arrow) in NAFLD model mice. (**d**) Volume of O_2 (VO_2) consumption, normalized to body weight. (**e**) Area under the curve for O_2 consumption. (**f**) Volume of CO_2 (VCO_2) expiration, normalized to body weight. (**g**) Area under the curve for CO_2 expiration. The black x-axis line segments represent the 12-h dark phases. (**h-o**) Plasma levels of AST, ALT, total cholesterol (T-CHO), and triglycerides (TG) were measured at 21 weeks and 31 weeks after starting the HFFCD diet (orange arrows). Data in b-c and e-o are presented as means ± SEM (d-g, $n = 4$; b-c and h-o, n =5 mice). P values were obtained from two-tailed unpaired t-tests. *$P < 0.05$, **$P < 0.01$, ***$P < 0.001$, ****$P < 0.0001$. Source data are provided as a Source Data file.

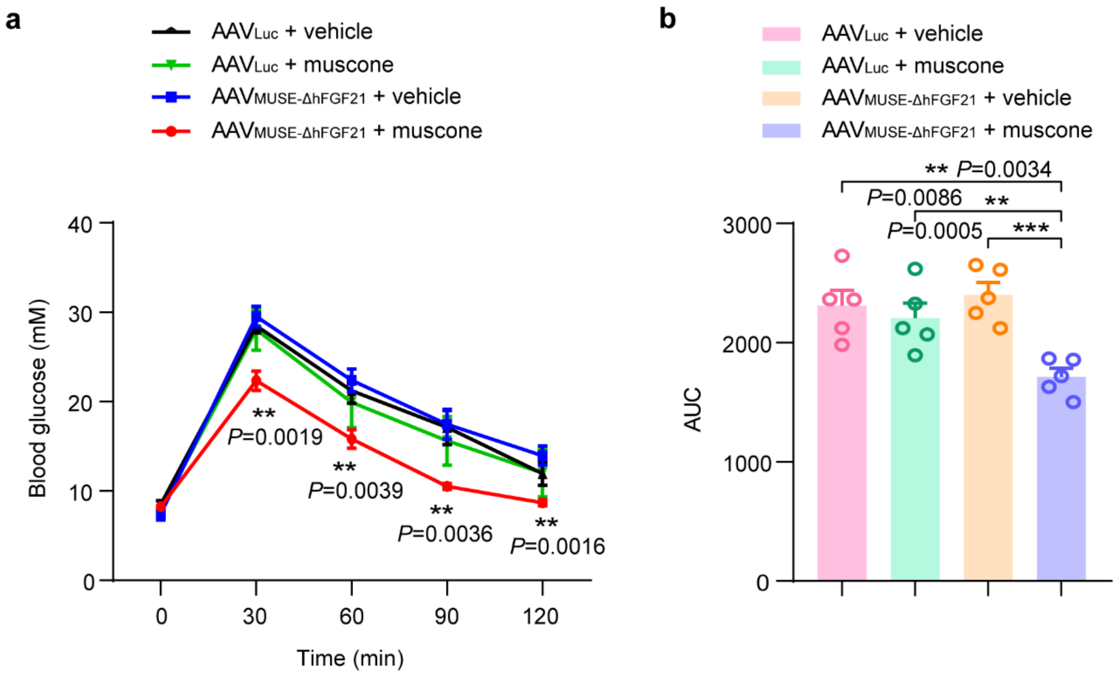

Supplementary Fig. 18 Intraperitoneal glucose tolerance test (IPGTT) in NAFLD model mice. (**a, b**) AAV_{MUSE}-mediated ΔhFGF21 production to control glucose homeostasis in NAFLD model mice. NAFLD model mice were transduced with $AAV_{MUSE\text{-}\Delta hFGF21}$ (2×10^{11} vg) or transduced with control AAV_{Luc} (2×10^{11} vg) via tail vein injection. One week after AAV injection, $AAV_{MUSE\text{-}\Delta hFGF21}$ transduced NAFLD model mice were exposed to nebulized muscone for 4 hours once each week until week 31. The examined controls included NAFLD model mice transduced with $AAV_{MUSE\text{-}\Delta hFGF21}$ and exposed to a vehicle (a mixture of castor oil and ddH_2O), and NAFLD model mice transduced with AAV_{Luc} with or without exposure to muscone. (**a**) IPGTT was performed by an intraperitoneal injection of D-glucose (1.5 g/kg body weight) at 22 weeks after starting the HFFCD diet. (**b**) Area under the curve (AUC) analysis of the IPGTT data from a. Data are presented as means ± SEM (n = 5 mice). P values were obtained from two-tailed unpaired t-tests. $^{**}P < 0.01$, $^{***}P < 0.001$. P values in (a) were calculated by comparing the $AAV_{MUSE\text{-}\Delta hFGF21}$-vehicle group with the $AAV_{MUSE\text{-}\Delta hFGF21}$-mucone group. Source data are provided as a Source Data file.

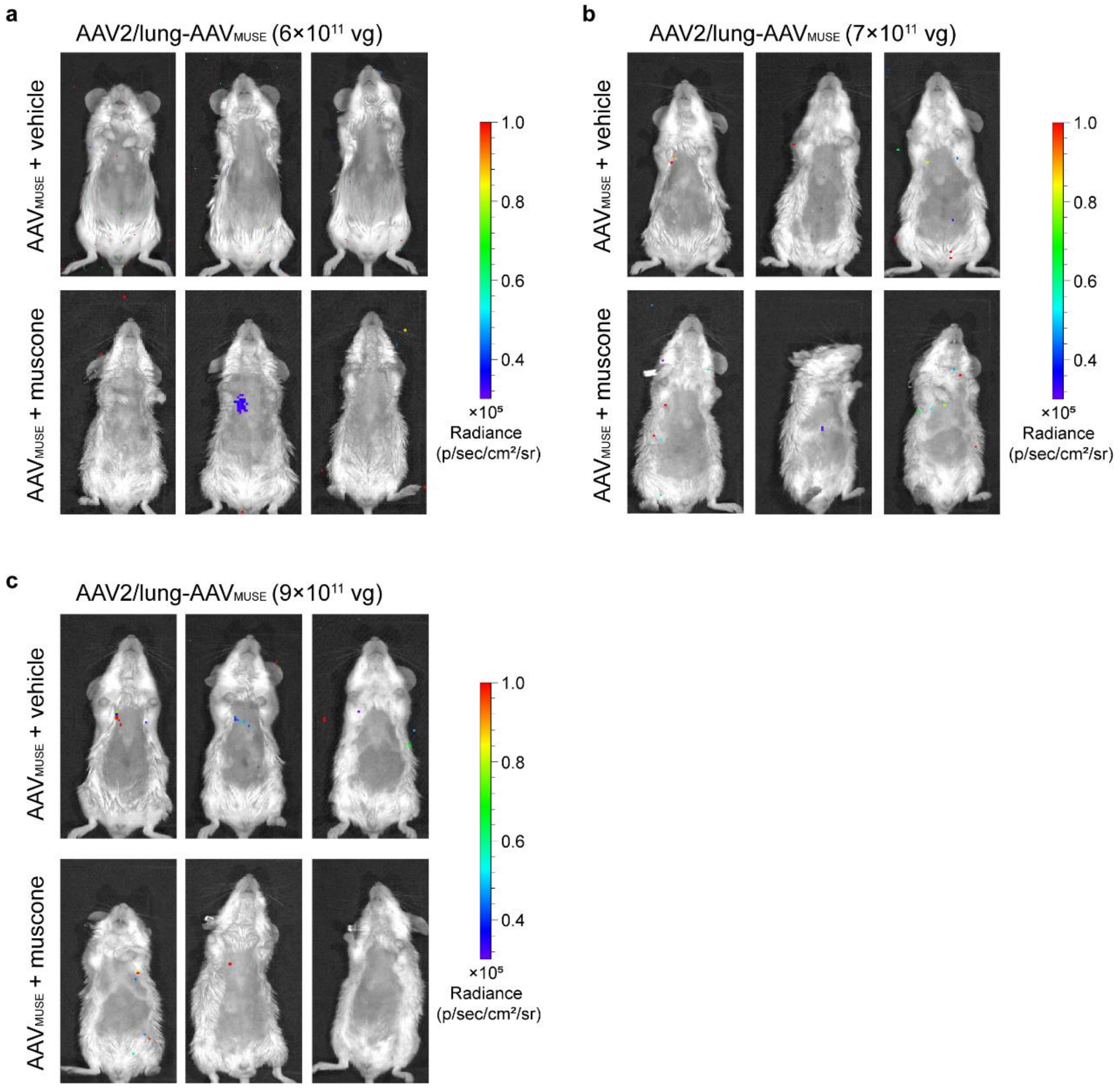

Supplementary Fig. 19 AAV2/lung-AAV$_{\text{MUSE}}$-mediated luciferase expression in mouse lungs. (**a-c**) *In vivo* bioluminescence images of the female BALB/c mice (8-week-old) transduced with different doses of AAV$_{\text{MUSE}}$ packaged into AAV2/lung capsid. (**a**) BALB/c mice were transduced with AAV$_{\text{MUSE}}$ containing three AAV2/lung vectors: pWX126 (ITR-P$_{\text{SV40}}$-MOR215-1-pA-ITR, 2×10^{11} vg), pWX127 (ITR-P$_{\text{SV40}}$-Gα_{olf}-P2A-RTP1S-pA-ITR, 2×10^{11} vg), and pWX158 (ITR-P$_{\text{CRE}}$-luciferase-P2A-EGFP-pA-ITR, 2×10^{11} vg) via tail vein injection. (**b**) BALB/c mice were transduced with AAV$_{\text{MUSE}}$ containing three AAV2/lung vectors: pWX126 (3×10^{11} vg), pWX127 (2×10^{11} vg), and pWX158 (2×10^{11} vg) via tail vein injection. (**c**) BALB/c mice were transduced with AAV$_{\text{MUSE}}$ containing three AAV2/lung vectors: pWX126 (3×10^{11} vg), pWX127 (3×10^{11} vg), and pWX158 (3×10^{11} vg) via tail vein injection. At 2 weeks after

the AAV injection, AAV_{MUSE}-transduced mice were exposed to a nebulized muscone or vehicle for 4 hours, and bioluminescence imaging was quantified 4 hours after exposure to nebulized muscone using an *in vivo* imaging system.

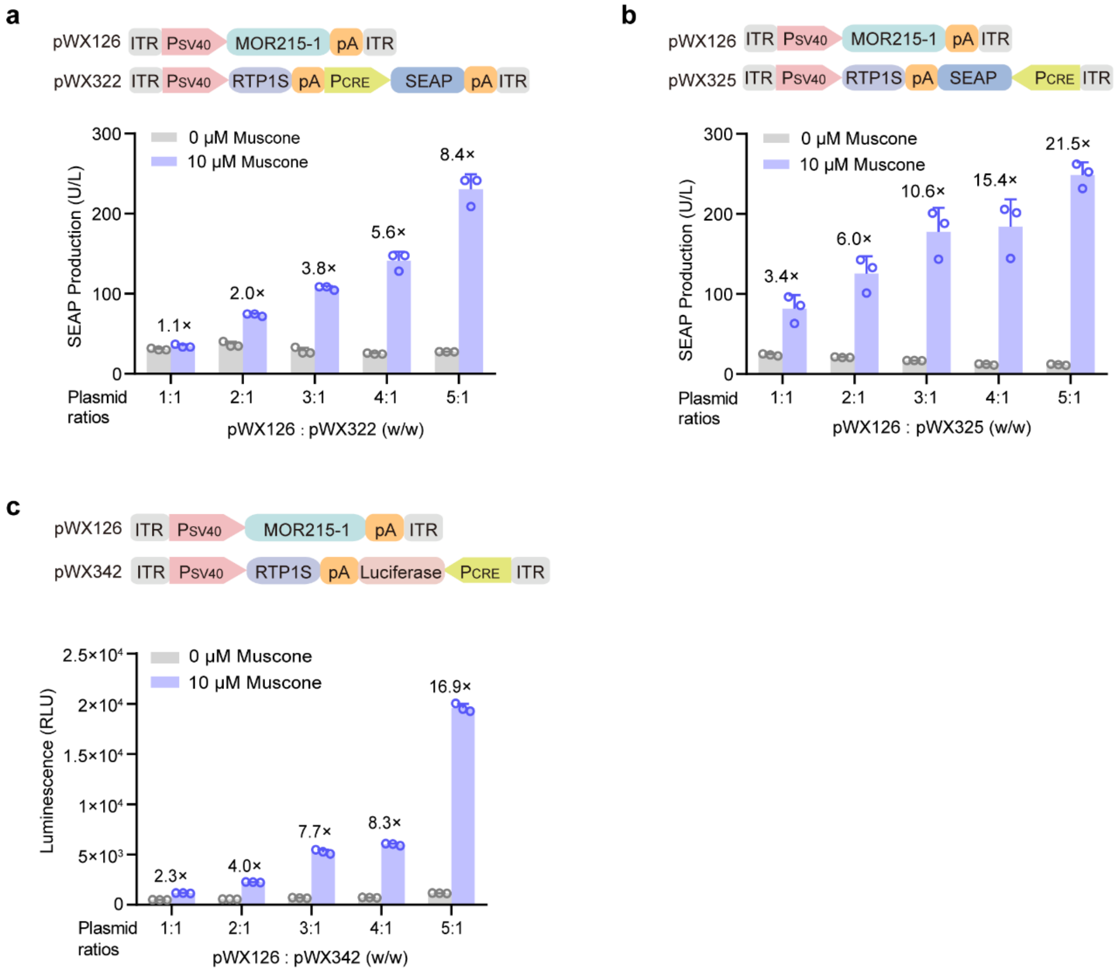

Supplementary Fig. 20 Construction and optimization of the AAV vector encoding AAV$_{\text{MUSE}}$ iteration in HEK-293T cells. (**a, b**) HEK-293T cells were co-transfected with pWX126 (ITR-P$_{\text{SV40}}$-MOR215-1-pA-ITR) and a concatenated vector for two expression cassettes (pWX322, ITR-P$_{\text{SV40}}$-RTP1S-pA::P$_{\text{CRE}}$-SEAP-pA-ITR or pWX325, ITR-P$_{\text{SV40}}$-RTP1S::pA-SEAP-P$_{\text{CRE}}$-ITR) at different ratios (w/w) and cultivated in culture medium with or without 10 μM muscone for 48 hours . SEAP production in the culture supernatant was profiled. (**c**) HEK-293T cells were co-transfected with pWX126 (ITR-P$_{\text{SV40}}$-MOR215-1-pA-ITR) and pWX342 (ITR-P$_{\text{SV40}}$-RTP1S::pA-luciferase-P$_{\text{CRE}}$-ITR) at different ratios (w/w) and cultivated in culture medium with or without 10 μM muscone. Luciferase levels were profiled 48 hours after muscone stimulation. RLU, relative light unit. Data are presented as means ± SD; *n* = 3 biologically independent samples. All plasmids are described in Supplementary Tables 1 and 3. Source data are provided as a Source Data file.

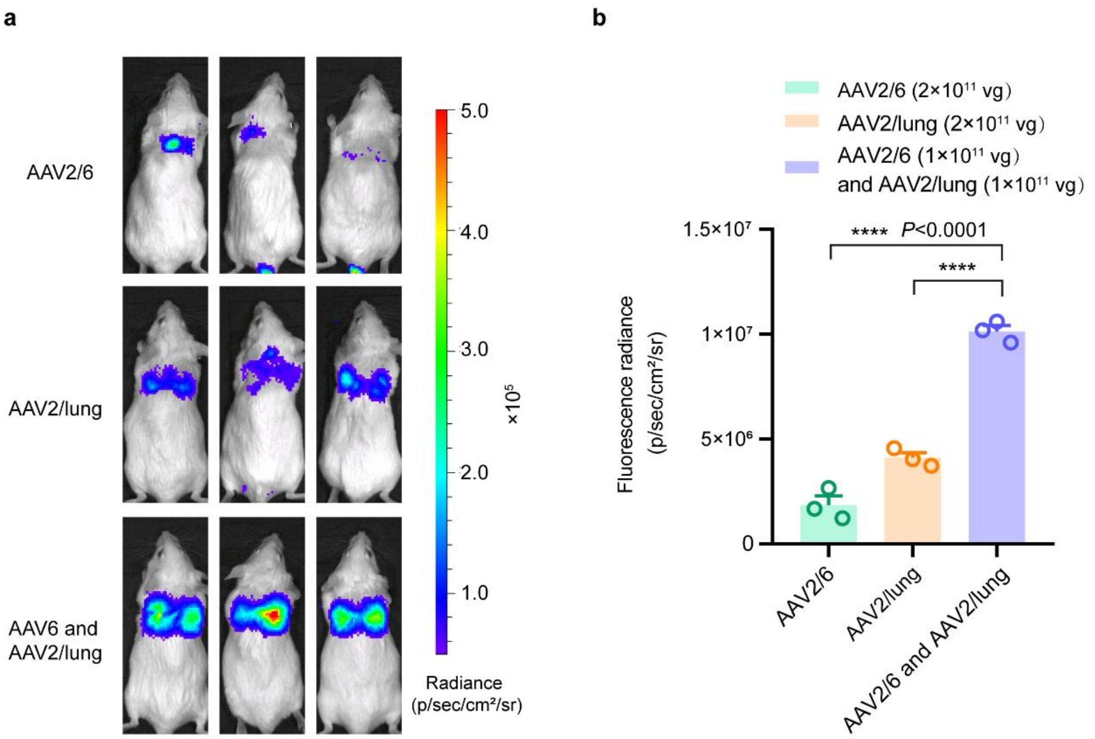

Supplementary Fig. 21 Assessing the impact of AAV serotype combinations on luciferase expression in mice. The female BALB/c mice (8-week-old) were transduced with luciferase reporter using AAV2/6 (2×10^{11} vg), or AAV2/lung (2×10^{11} vg), or dual AAV serotype combination (AAV2/6, 1×10^{11} vg and AAV2/lung, 1×10^{11} vg) by tail vein injection. (**a**) *In vivo* bioluminescence images of the BALB/c mice transduced with three different AAV vectors packaging luciferase (ITR-P_{CMV}-luciferase-pA-ITR) at 2 weeks after the AAV injection. (**b**) Bioluminescence measurements of the luciferase expression based on bioluminescence imaging in a. Data in b are presented as means ± SEM ($n = 3$ mice). P values were obtained from two-tailed unpaired t-tests. ****$P < 0.0001$. Source data are provided as a Source Data file.

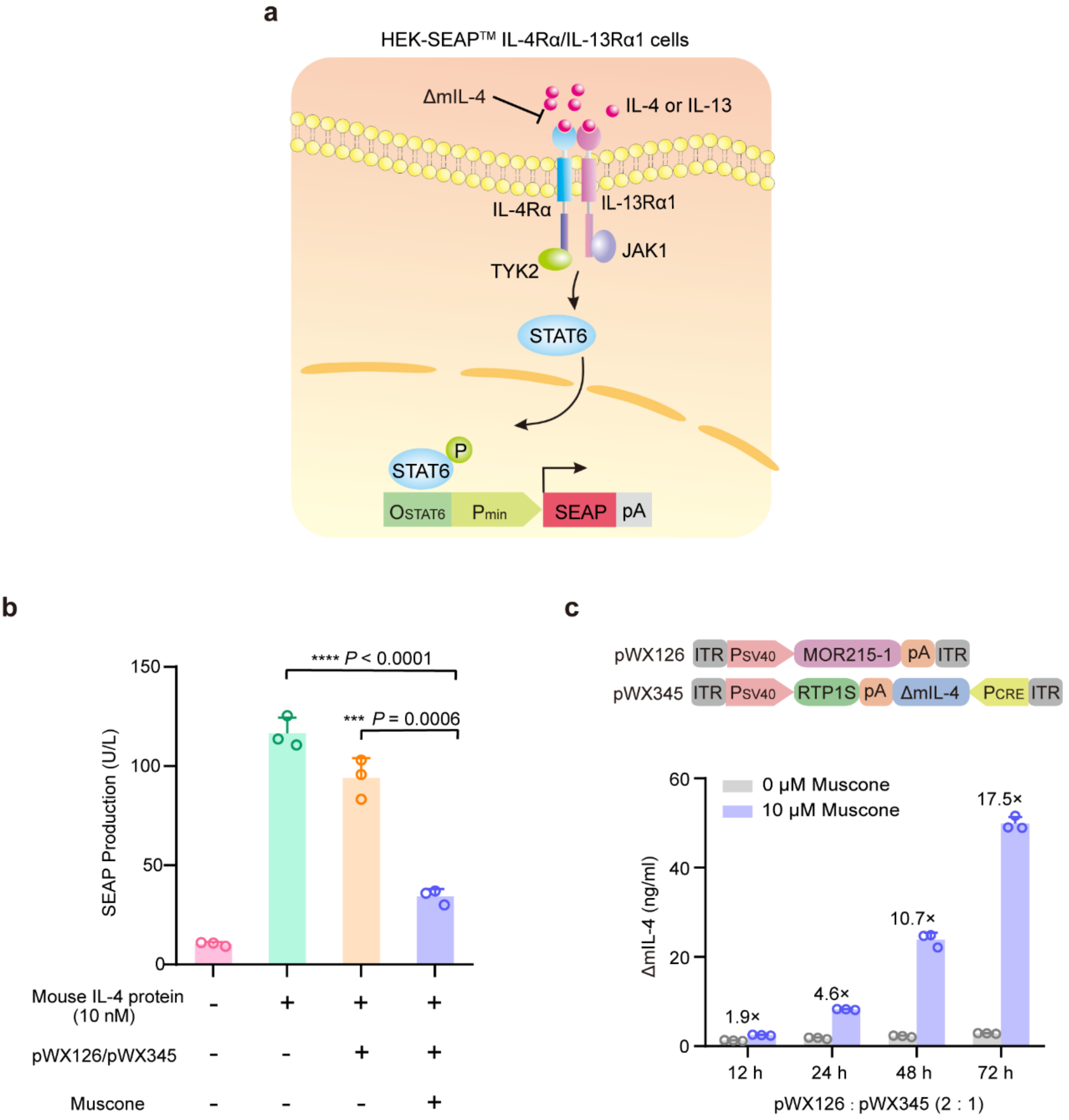

Supplementary Fig. 22 Validation of the biological activity of ΔmIL-4 and characterization of the kinetics of AAV$_{MUSE}$-mediated ΔmIL-4 expression. (**a**) Schematic representation of an engineered HEK-SEAP™ IL-4Rα/IL-13Rα1 cell line, which was used to verify the biological activity of AAV$_{MUSE}$-induced ΔmIL-4 [a variant of the murine IL-4 protein with double mutations (Q116D, Y119D)]. When ΔmIL-4 bound to the IL-4Rα/IL-13α1 heterodimeric receptor complex, the cellular IL-4/IL-13 signaling pathway and tyrosine phosphorylation of the transcription factor STAT6 were blocked, inhibiting P$_{STAT6}$-driven SEAP reporter expression. (**b**) HEK-293T cells were co-transfected with pWX126 and pWX345 and cultivated in culture medium with or without 10 μM muscone for 24 hours. Then HEK-SEAP™ IL-4Rα/IL-13Rα1 cells were

co-transfected with an interleukin-4 receptor α subunit plasmid pIL-4Rα (P_{CMV}-IL-4Rα-pA; 100 ng), an interleukin-13 receptor α1 subunit plasmid pIL-13Rα1 (P_{CMV}-IL-13Rα1-pA; 100 ng), a mouse signal transducer and activator of transcription 6 plasmid pSTAT6 (ITR-P_{CMV}-mSTAT6::P_{PGK}-puromycin-ITR; 20 ng), and a STAT6-inducible SEAP reporter plasmid pWX326 (P_{STAT6}-SEAP-pA; $P_{STAT6,}$ O_{STAT6}-P_{min}; 100 ng), and cultivated in supernatant from pWX126/pWX345-cotransfected HEK-293T cells and supplemented with mIL-4 (10 nM), or in mIL-4 alone (10 nM), or in culture medium without mIL-4. SEAP production in the culture supernatant was profiled after 24 hours. (**c**) Muscone exposure time-dependent AAV_{MUSE}-mediated ΔmIL-4 expression kinetics. HEK-293T cells were co-transfected with pWX126/pWX345 and cultivated for the indicated periods (0 to 72 hours) with or without 10 μM muscone. ΔmIL-4 levels in the culture supernatant were quantified at the indicated time points (X-axis, 12 to 72 hours). Data are presented as means ± SD; n = 3 biologically independent samples. P values were obtained from two-tailed unpaired t-tests. ***$P < 0.001$, ****$P < 0.0001$. Descriptions of all plasmids, and detailed descriptions of the genetic constructs, are provided in Supplementary Tables 1 and 3. Source data are provided as a Source Data file.

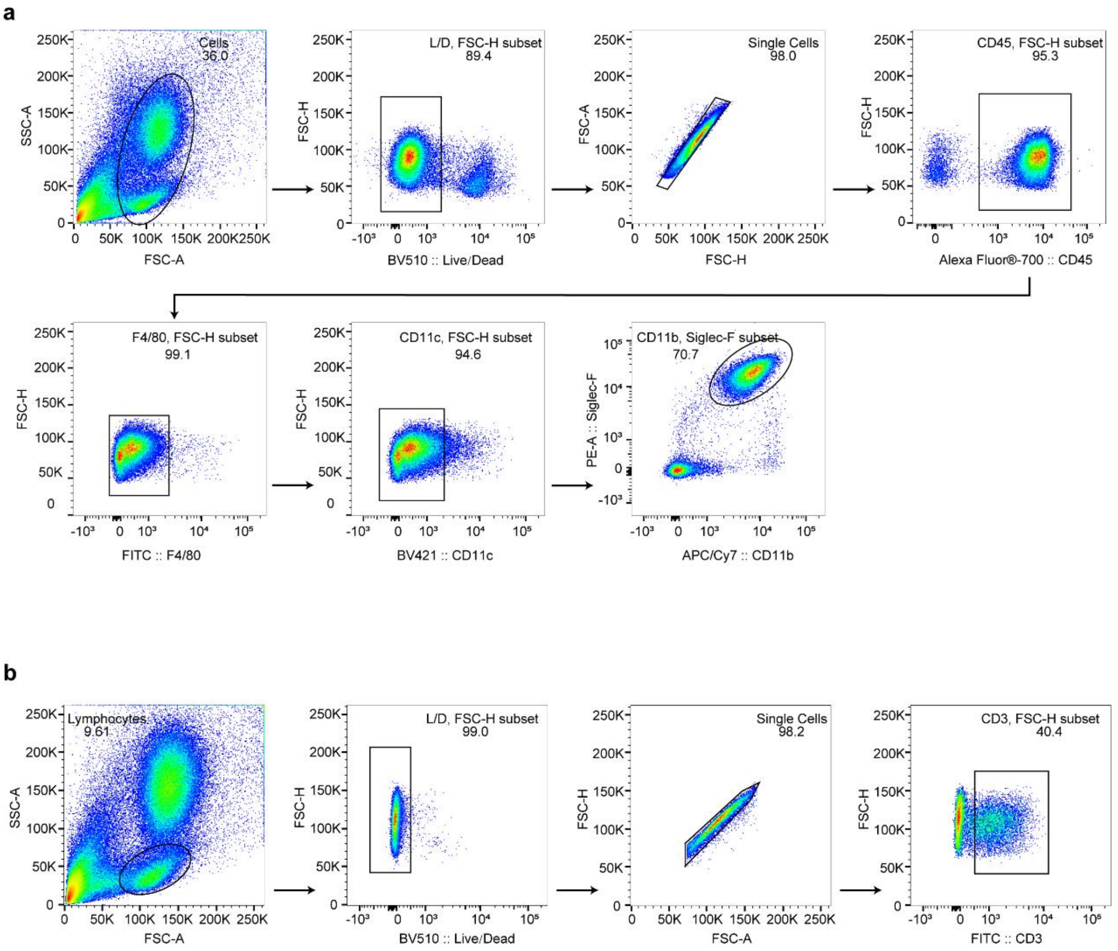

Supplementary Fig. 23 Representative flow cytometry staining of eosinophils and T cells obtained from the BALF and lungs of mice. Eosinophils and T cells were gated on viability-dye negative cells to exclude dead cells, and doublets were excluded based on size (FSC) and granularity (SSC). (**a**) Expression of CD11b^{+}siglec-F^{+} was used to define eosinophil cells. (**b**) Expression of CD3^{+} was used to determine T cells. Representative results of one independent experiment (*n* = 5 mice per group).

Table S1. Plasmids designed and used in this study.

Plasmids	Description and cloning strategy	Reference
pcDNA3.1 (+)	Constitutive mammalian P_{hCMV}-driven expression vector (P_{hCMV}-MCS-pA)	Invitrogen, CA
pSEAP2-control	Constitutive mammalian P_{SV40}-driven SEAP expression vector (P_{SV40}-SEAP-pA).	Clontech, CA
pCK53	A synthetic cAMP-responsive promoter (P_{CRE}) - driven SEAP expression vector (P_{CRE}-SEAP-pA).	Kemmer, C. et al[1]
pGα_{olf}	Constitutive mammalian P_{SV40}-driven olfactory neuron-specific G protein alpha subunit (Gα_{olf}) expression vector (P_{SV40}-Gα_{olf}-pA).	Shirasu, M. et al[2]
pMOR215-1	Constitutive mammalian P_{SV40}-driven muscone-responsive G-protein-coupled receptor MOR215-1 expression vector (P_{SV40}-MOR215-1-pA).	Shirasu, M. et al[2]
pMF111	Tetracycline-responsive SEAP expression vector ($P_{hCMV*-1}$-SEAP-pA; $P_{hCMV*-1}$, O_{TetO7}-$P_{hCMVmin}$).	Fussenegger, M. et al[3]
pRTP1S	Constitutive mammalian P_{SV40}-driven truncated receptor-transporting protein 1 (RTP1S) expression vector (P_{SV40}-RTP1S-pA)	Dahoun, T. et al[4]
pTetR-ELK1	Constitutive mammalian P_{CMV}-driven synthetic hybrid transcription factor TetR-ELK1 expression vector (P_{CMV}-TetR-ELK1-pA).	Keeley, M.B. et al[5]
pIR	Constitutive mammalian P_{CMV}-driven human IR expression vector (P_{hCMV}-hIR-pA; IR, insulin receptor).	Addgene (no. 24049)
pTRPM8	Constitutive mammalian P_{CMV}-driven human TRPM8 expression vector (P_{hCMV}-hTRPM8-pA; TRPM8, transient receptor potential (TRP) melastatin 8).	Bai, P. et al[6]
pTLR2	Constitutive mammalian P_{CMV}-driven human TLR2 expression vector (P_{hCMV}-hTLR2-pA; TLR2, toll-like receptor 2).	Liu, Y. et al[7]
pMX57	P_{NFAT3}-driven SEAP expression vector (P_{NFAT3}-SEAP-pA; P_{NFAT3}, $(NFAT_{IL4})_5$-P_{min}).	Xie, M. et al[8]
pYW27	$P_{NF\text{-}\kappa B}$-driven SEAP expression vector ($P_{NF\text{-}\kappa B}$-SEAP-pA).	This work
pKLB	Constitutive mammalian P_{CMV}-driven Klotho beta expression vector (P_{CMV}-βKlotho-pA).	This work

pIL-4Rα	Constitutive mammalian P_{hCMV}-driven mouse interleukin-4 receptor α subunit expression vector (P_{hCMV}-IL-4Rα-pA).	This work
pIL-13Rα1	Constitutive mammalian P_{hCMV}-driven mouse interleukin-13 receptor α1 subunit expression vector (P_{hCMV}-IL-13Rα1-pA).	This work
pSTAT6	Constitutive mammalian P_{hCMV}-driven mouse signal transducer and activator of transcription 6 (STAT6) expression vector (ITR-P_{hCMV}-mSTAT6::P_{PGK}-puromycin-ITR).	This work
pWX126	AAV vector carrying constitutive P_{SV40}-driven expression unit for MOR215-1 (ITR-P_{SV40}-MOR215-1-pA-ITR).	This work
pWX127	AAV vector carrying constitutive P_{SV40}-driven expression units for concatenated $G\alpha_{olf}$ and RTP1S (ITR-P_{SV40}-$G\alpha_{olf}$-P2A-RTP1S-pA-ITR).	This work
pWX158	AAV vector carrying P_{CRE}-driven expression unit for luciferase and EGFP (ITR-P_{CRE}-luciferase-P2A-EGFP-pA-ITR).	This work
pWX252	AAV vector carrying P_{CRE}-driven expression unit for ΔhFGF21 [ITR-P_{CRE}-ΔhFGF21-pA-ITR; ΔhFGF21, the fusion protein of hFc and hFGF21 with three mutations (L98R, P171G, and A180E)].	This work
pWX322	AAV vector carrying a constitutive P_{SV40}-driven RTP1S expression unit and a P_{CRE}-driven SEAP expression unit in the same direction (ITR-P_{SV40}-RTP1S-pA::P_{CRE}-SEAP-pA-ITR).	This work
pWX325	AAV vector carrying a constitutive P_{SV40}-driven RTP1S expression unit and a P_{CRE}-driven SEAP expression unit in the converse direction (ITR-P_{SV40}-RTP1S::pA-SEAP-P_{CRE}-ITR).	This work
pWX326	A STAT6-inducible SEAP reporter vector (P_{STAT6}-SEAP-pA; P_{STAT6}, O_{STAT6}-P_{min} containing STAT6-binding sites 5′ of a minimal promoter)	This work
pWX342	AAV vector carrying a constitutive P_{SV40}-driven RTP1S expression unit and a P_{CRE}-driven luciferase expression unit (ITR-P_{SV40}-RTP1S::pA-luciferase-P_{CRE}-ITR).	This work
pWX345	AAV vector carrying a constitutive P_{SV40}-driven RTP1S expression unit and a P_{CRE}-driven ΔmIL-4 expression unit [(ITR-P_{SV40}-RTP1S::pA-ΔmIL-4-P_{CRE}-ITR; ΔmIL-4, IL-4 introducing two mutants (Q116D and Y119D)].	This work

Supplementary Note 1. Abbreviations

AAV, adeno-associated virus; **ITR**, inverted terminal repeat; **pA**, polyadenylation signal; **P_{hCMV}**, human cytomegalovirus immediate early promoter; **2A**, self-cleaving peptide engineered for bicistronic gene expression in mammalian cells; **P2A:** porcine teschovirus-1 2A; **TetR**, *Escherichia coli* Tn10-derived tetracycline-dependent repressor of the tetracycline resistance gene; **ELK1**, human ETS domain-containing transcription factor Elk1; **SEAP**, human placental secreted alkaline phosphatase; **P_{SV40}**, simian virus 40 promoter; **$P_{hCMV*-1}$**, tetracycline-responsive promoter (O_{tetO7}-$P_{hCMVmin}$); **P_{min},** minimal eukaryotic TATA-box promoter (5'-TAGAGGGTATATAATGGAAGCTCGACTTC CAG-3'); **MCS**, multiple cloning site; **$G\alpha_{olf}$**, olfactory neuron-specific G protein α subunit; **MOR**, *Mus musculus* olfactory receptors; **RTP1S**, short version of receptor transport protein 1; **CRE**, cAMP-responsive element (CREB1 binding site); **P_{CRE}**, synthetic cAMP-responsive mammalian promoter containing three CRE repeats; **Luc**, firefly luciferase; **IL-4Rα**, interleukin-4 receptor alpha subunit; **IL-13Rα1**, interleukin-13 receptor alpha 1 subunit; **STAT6**, signal transducer and activator of transcription 6 (mouse); **ΔmIL-4**, a variant of the murine interleukin 4 (IL-4) introducing two mutations (Q116D and Y119D); **hFc_{IgG1},** a human immunoglobulin-G1 (IgG1) fragment crystallizable (Fc) domain; **ΔhFGF21**, a fusion protein stabilized by fusing a hFc with a human signal peptide to the N terminus of FGF21, which contained three mutations (L98R, P171G, and A180E); **SP**, signal peptide.

Table S2. Oligonucleotide sequences used for qPCR analysis.

Gene name	Primer name	Primer sequence (5'-3')
Luciferase	Forward	ACACCCGAGGGGGATGATAA
	Reverse	GTGTTCGTCTTCGTCCCAGT

Table S3. Detailed DNA sequence information

1. pWX126: ITR-P_{SV40}-MOR215-1-pA-ITR

CCTGCAGGCAGCTGCGCGCTCGCTCGCTCACTGAGGCCGCCCGGGCAAAGCCCGGG<br>CGTCGGGCGACCTTTGGTCGCCCGGCCTCAGTGAGCGAGCGAGCGCGCAGAGAGGG<br>AGTGGCCAACTCCATCACTAGGGGTTCCTGCGGCCGCTAGCATGCATCTCAATTAG<br>TCAGCAACCATAGTCCCGCCCCTAACTCCGCCCATCCCGCCCCTAACTCCGCCCAG<br>TTCCGCCCATTCTCCGCCCCATGGCTGACTAATTTTTTTTATTTATGCAGAGGCCG<br>AGGCCGCCTCGGCCTCTGAGCTATTCCAGAAGTAGTGAGGAGGCTTTTTTGGAGGC<br>CTAGGCTTTTGCAAAAAGCTCCTCCGATCGAGGGGCTCGCATCTCTCCTTCACGCG<br>CCCGCCGCCCTACCTGAGGCCGCCATCCACGCCGGTTGAGTCGCGTTCTGCCGCCT<br>CCCGCCTGTGGTGCCTCCTGAACTGCGTCCGCCGTCTAGGTAAGTTTAAAGCTCAG<br>GTCGAGACCGGGCCTTTGTCCGGCGCTCCCTTGGAGCCTACCTAGACTCAGCCGGC<br>TCTCCACGCTTTGCCTGACCCTGCTTGCTCAACTCTACGTCTTTGTTTCGTTTTCT<br>GTTCTGCGCCGTTACAGATCCAAGCTCTGAAAAACCAGAAAGTTAACTGGTAAGTT<br>TAGTCTTTTTGTCTTTTATTTCAGGTCCCGGATCCGGTGGTGCAAATCAAAGAACT<br>GCTCCTCAGTGGATGTTGCCTTTACTTCTAGGCCTGTACGGAAGTGTTACTTCTGC<br>TCTAAAAGCTGCGGAATTGGCCACCATGAACGGGACCGAGGGCCCAAACTTCTACG<br>TGCCTTTCTCCAACAAGACGGGCGTGGTGGAATTCGCCACCATGCCTGGAGGGAGG<br>AATAGCACAGTCATCACCAAGTTCATCCTTGTGGGATTCTCAGATTTTCCAAAGCT<br>CAAGCTGGTTCTCTTTGTTATCTTCCTGGGAAGTTATCTCTCCACAGTGGTGTGGA<br>ACTTGGGCCTCATCATCTTGATTAGGATTGACCCTTACCTACACACACCTATGTAC<br>TTCTTCCTCAGCAATTTGTCATTTTTAGATTTCTGTTACATTTCATCTACAACCCC<br>TAAAATGCTCTCGGGATTCTTCCAGAAGTCTAAATCTATCTCCTTTGTTGGGTGCA<br>CCATGCAGTACTTCATCTTCTCAAGCCTGGGTCTGTCCGAATGCTGCCTTCTGGCA<br>GCCATGGCTTATGACCGGTATGCTGCCATTTGTAATCCTCTTCTCTACACAGCCAT<br>CATGTCCCCGTCACTCTGTGTGCACATGGTGGTTGGAGCCTATAGTACTGGTCTCT<br>TGGGTTCATTGATTCAACTGTGTGCTATACTTCAGCTCCATTTCTGTGGGCCAAAT<br>ATTATAAACCATTTCTTTTGTGACCTGCCTCAGCTATTAGTTCTTTCCTGCTCTGA<br>AACCTTTCCCCTGCAAGTCTTGAAATTTGTAATAGCAGTGATTTTTGGGGTGGCAT<br>CTGTCATTGTTATCCTGATATCCTATGGTTATATCATTGGCACAATCCTGAATATC<br>AGCTCAGTAGAAGGTAGGTCCAAGGCATTCAATACCTGTGCCTCTCACCTGACAGC<br>AGTCACCCTCTTTTTTGGATCAGGACTCTTTGTCTATATGCGCCCCAGCTCCAACA<br>GTTCCCAGGGTTATGACAAGATGGCTTCCGTGTTCTATACAGTGGTGATTCCCATG<br>TTGAATCCTCTGATTTATAGTCTCAGGAACAAGGAAATAAAAGATGCTCTTCAGAG<br>ATGTAAAAATAAGTGCTTTTCTCAGTGCCACTGTTAAAGCGGCGCCAGCGGCCGCT<br>CTAGACAGACATGATAAGATACATTGATGAGTTTGGACAAACCACAACTAGAATGC<br>AGTGAAAAAAATGCTTTATTTGTGAAATTTGTGATGCTATTGCTTTATTTGTAACC<br>ATTATAAGCTGCAATAAACAAGTTAACAACAACAATTGCATTCATTTTATGTTTCA<br>GGTTCAGGGGGAGGTGTGGGAGGTTTTTTAAAGCAAGTAAAACCTCTACAAATGTG<br>GTATGGGAGCTCGCGGCCGCAGGAACCCCTAGTGATGGAGTTGGCCACTCCCTCTC<br>TGCGCGCTCGCTCGCTCACTGAGGCCGGGCGACCAAAGGTCGCCCGACGCCCGGGC<br>TTTGCCCGGGCGGCCTCAGTGAGCGAGCGAGCGCGCAGCTGCCTGCAGG

2. pWX127: ITR-P_{SV40}-$G\alpha_{olf}$-P2A-RTP1S-pA-ITR

CCTGCAGGCAGCTGCGCGCTCGCTCGCTCACTGAGGCCGCCCGGGCAAAGCCCGGG
CGTCGGGCGACCTTTGGTCGCCCGGCCTCAGTGAGCGAGCGAGCGCGCAGAGAGGG
AGTGGCCAACTCCATCACTAGGGGTTCCTGCGGCCGCTAGCATGCATCTCAATTAG
TCAGCAACCATAGTCCCGCCCCTAACTCCGCCCATCCCGCCCCTAACTCCGCCCAG
TTCCGCCCATTCTCCGCCCCATGGCTGACTAATTTTTTTTATTTATGCAGAGGCCG
AGGCCGCCTCGGCCTCTGAGCTATTCCAGAAGTAGTGAGGAGGCTTTTTTGGAGGC
CTAGGCTTTTGCAAAAAGCTCCTCCGATCGAGGGGCTCGCATCTCTCCTTCACGCG
CCCGCCGCCCTACCTGAGGCCGCCATCCACGCCGGTTGAGTCGCGTTCTGCCGCCT
CCCGCCTGTGGTGCCTCCTGAACTGCGTCCGCCGTCTAGGTAAGTTTAAAGCTCAG
GTCGAGACCGGGCCTTTGTCCGGCGCTCCCTTGGAGCCTACCTAGACTCAGCCGGC
TCTCCACGCTTTGCCTGACCCTGCTTGCTCAACTCTACGTCTTTGTTTCGTTTTCT
GTTCTGCGCCGTTACAGATCCAAGCTCTGAAAAACCAGAAGTTAACTGGTAAGTT
TAGTCTTTTTGTCTTTTATTTCAGGTCCCGGATCCGGTGGTGCAAATCAAAGAACT
GCTCCTCAGTGGATGTTGCCTTTACTTCTAGGCCTGTACGGAAGTGTTACTTCTGC
TCTAAAAGCTGCGGAATTGGCCACCATGAACGGGACCGAGGGCCCAAACTTCTACG
TGCCTTTCTCCAACAAGACGGGCGTGGTGGAATTCGCCACCATGGGGTGTTTGGGC
AACAGCAGCAAGACCGCGGAAGAAGATCAGGGCGTAGATGAAAAAGAACGCCGCGA
GGCCAACAAAAAGATCGAGAAGCAGTTGCAGAAAGAGCGCCTGGCTTACAAAGCAA
CTCACCGCCTGCTGCTTCTGGGGGCTGGTGAGTCCGGGAAAAGCACTATCGTCAAA
CAGATGAGGATCCTGCACGTCAATGGCTTCAACCCCGAGGAAAAGAAGCAGAAAAT
TCTGGACATCAGGAAAAATGTCAAAGATGCGATCGTGACAATCGTTTCAGCAATGA
GTACTATCATACCTCCAGTTCCACTGGCCAACCCTGAGAACCAGTTCCGGTCAGAT
TATATCAAGAGCATAGCCCCTATCACTGACTTTGAATATTCCCAGGAGTTCTTTGA
CCATGTGAAGAAGCTGTGGGACGATGAAGGAGTGAAGGCCTGCTTTGAGAGATCCA
ACGAGTACCAGCTGATCGACTGTGCACAATACTTCCTGGAAAGGATTGACAGTGTC
AGTCTGGTTGACTACACACCCACAGACCAGGACCTGCTCAGATGCAGAGTGCTGAC
ATCAGGAATCTTTGAGACACGATTCCAAGTGGACAAAGTGAACTTTCACATGTTTG
ATGTTGGAGGCCAGAGAGATGAGAGAAGAAAATGGATCCAGTGTTTTAATGATGTC
ACTGCGATCATTTACGTGGCGGCCTGTAGTAGCTACAACATGGTGATCCGGGAAGA
TAACAATACCAACAGACTTCGGGAATCACTGGACCTGTTTGAAAGCATCTGGAATA
ACAGGTGGTTGCGAACCATTTCTATCATCCTATTCTTGAACAAACAAGACATGCTG
GCAGAAAAAGTCTTGGCAGGGAAGTCAAAAATCGAAGACTATTTCCCGGAGTATGC
CAATTATACTGTCCCTGAAGATGCAACACCAGATGCGGGAGAAGATCCCAAAGTTA
CAAGAGCAAAGTTCTTTATCCGGGATCTGTTCTTGAGGATCAGCACAGCCACGGGT
GATGGCAAACATTACTGCTACCCTCACTTCACCTGCGCCGTGGACACAGAGAACAT
CCGCAGAGTGTTCAACGATTGCCGTGACATCATCCAGAGAATGCATCTCAAGCAGT
ACGAACTCTTGGCCACGAACTTCTCTCTGTTAAAGCAAGCAGGAGATGTTGAAGAA
AACCCCGGGCCCATGTGTAAGAGTGTGACCACAGGTGAGTGGAAGAAGGTCTTCTA
CGAAAAGATGGAGGAGGTGAAGCCAGCGGACAGCTGGGACTTCATCATAGACCCCA
ACCTCAAGCACAATGTGTTGGCCCCTGGCTGGAAGCAGTACCTGGAACTTCATGCC
TCAGGCAGGTTCCACTGTTCCTGGTGCTGGCACACCTGGCAGTCACCCCATGTAGT
CATCCTCTTCCACATGTACCTGGACAAGGCTCAGCGCGCTGGTTCGGTGCGCATGC
GTGTGTTCAAGCAGCTCTGCTACGAGTGCGGTACAGCACGGCTGGATGAGTCCAGC
ATGCTGGAGGAGAACATCGAAAGCCTGGTGGACAACCTCATCACCAGTTTGCGAGA

```
GCAGTGCTACGGGGAGCGTGGTGGCCACTACCGCATCCATGTGGCCAGCCGGCAGG
ACAACCGGCGACACCGCGGAGAGTTCTGCGAGGCCTGCCAGGAAGGCATCGTGCAC
TGGAAGCCCAGTGAGAAGCTGCTGGAGGAGGAGGCGACCACCTACACCTTCTCCCG
TGCTCCCAGCCCCACCAAACCGCAGGCTGAAACAGGCTCAGGCTGCAACTTCTGCT
CCATTCCCTGGTGCTTATTTGGGCCACGGTTTGATGCTCATCATCTACCTGCAA
TTCTCCTTCCGTACTTCTGTCTAACTCGAGGGATCTTCCATACCTACCTCTAGACA
GACATGATAAGATACATTGATGAGTTTGGACAAACCACAACTAGAATGCAGTGAAA
AAAATGCTTTATTTGTGAAATTTGTGATGCTATTGCTTTATTTGTAACCATTATAA
GCTGCAATAAACAAGTTAACAACAACAATTGCATTCATTTTATGTTTCAGGTTCAG
GGGGAGGTGTGGGAGGTTTTTTAAAGCAAGTAAAACCTCTACAAATGTGGTATGGG
AGCTCGCGGCCGCAGGAACCCCTAGTGATGGAGTTGGCCACTCCCTCTCTGCGCGC
TCGCTCGCTCACTGAGGCCGGGCGACCAAAGGTCGCCCGACGCCCGGGCTTTGCCC
GGGCGGCCTCAGTGAGCGAGCGAGCGCGCAGCTGCCTGCAGG
```

3. pWX158: ITR-P_{CRE}-luciferase-P2A-EGFP-pA-ITR

```
CCTGCAGGCAGCTGCGCGCTCGCTCGCTCACTGAGGCCGCCCGGGCAAAGCCCGGG
CGTCGGGCGACCTTTGGTCGCCCGGCCTCAGTGAGCGAGCGAGCGCGCAGAGAGGG
AGTGGCCAACTCCATCACTAGGGGTTCCTGCGGCCGCTAGCGCAATAAAATATCTT
TATTTTCATTACATCTGTGTGTTGGTTTTTTGTGTGAATCGATAGTACTAACATAC
GCTCTCCATCAAAACAAAACGAAACAAAACAAACTAGCAAAATAGGCTGTCCCCAG
TGCAAGTGCAGGTGCCAGAACATTTCTCTATCGATAGGTACCGAGCTCTTACGCGT
GCTAGCGCACCAGACAGTGACGTCAGCTGCCAGATCCCATGGCCGTCATACTGTGA
CGTCTTTCAGACACCCCATTGACGTCAATGGGAGAACAGATCTGCCGCCCCGACTG
CATCTGCGTGTTCGAATTCGCCAATGACAAGACGCTGGGCGGGGTTTGTGTCATCA
TAGAACTAAAGACATGCAAATATATTTCTTCCGGGGACACCGCCAGCAAACGCGAG
CAACGGGCCACGGGGATGAAGCAGAAGCTTGCCACCATGGAAGACGCCAAAAACAT
AAAGAAAGGCCCGGCGCCATTCTATCCGCTGGAAGATGGAACCGCTGGAGAGCAAC
TGCATAAGGCTATGAAGAGATACGCCCTGGTTCCTGGAACAATTGCTTTTACAGAT
GCACATATCGAGGTGGACATCACTTACGCTGAGTACTTCGAAATGTCCGTTCGGTT
GGCAGAAGCTATGAAACGATATGGGCTGAATACAAATCACAGAATCGTCGTATGCA
GTGAAAACTCTCTTCAATTCTTTATGCCGGTGTTGGGCGCGTTATTTATCGGAGTT
GCAGTTGCGCCCGCGAACGACATTTATAATGAACGTGAATTGCTCAACAGTATGGG
CATTTCGCAGCCTACCGTGGTGTTCGTTTCCAAAAAGGGGTTGCAAAAAATTTTGA
ACGTGCAAAAAAAGCTCCCAATCATCCAAAAAATTATTATCATGGATTCTAAAACG
GATTACCAGGGATTTCAGTCGATGTACACGTTCGTCACATCTCATCTACCTCCCGG
TTTTAATGAATACGATTTTGTGCCAGAGTCCTTCGATAGGGACAAGACAATTGCAC
TGATCATGAACTCCTCTGGATCTACTGGTCTGCCTAAAGGTGTCGCTCTGCCTCAT
AGAACTGCCTGCGTGAGATTCTCGCATGCCAGAGATCCTATTTTTGGCAATCAAAT
CATTCCGGATACTGCGATTTTAAGTGTTGTTCCATTCCATCACGGTTTTGGAATGT
TTACTACACTCGGATATTTGATATGTGGATTTCGAGTCGTCTTAATGTATAGATTT
GAAGAAGAGCTGTTTCTGAGGAGCCTTCAGGATTACAAGATTCAAAGTGCGCTGCT
GGTGCCAACCCTATTCTCCTTCTTCGCCAAAAGCACTCTGATTGACAAATACGATT
TATCTAATTTACACGAAATTGCTTCTGGTGGCGCTCCCCTCTCTAAGGAAGTCGGG
```

GAAGCGGTTGCCAAGAGGTTCCATCTGCCAGGTATCAGGCAAGGATATGGGCTCAC
TGAGACTACATCAGCTATTCTGATTACACCCGAGGGGGATGATAAACCGGGCGCGG
TCGGTAAAGTTGTTCCATTTTTTGAAGCGAAGGTTGTGGATCTGGATACCGGGAAA
ACGCTGGGCGTTAATCAAAGAGGCGAACTGTGTGTGAGAGGTCCTATGATTATGTC
CGGTTATGTAAACAATCCGGAAGCGACCAACGCCTTGATTGACAAGGATGGATGGC
TACATTCTGGAGACATAGCTTACTGGGACGAAGACGAACACTTCTTCATCGTTGAC
CGCCTGAAGTCTCTGATTAAGTACAAAGGCTATCAGGTGGCTCCCGCTGAATTGGA
ATCCATCTTGCTCCAACACCCCAACATCTTCGACGCAGGTGTCGCAGGTCTTCCCG
ACGATGACGCCGGTGAACTTCCCGCCGCCGTTGTTGTTTTGGAGCACGGAAAGACG
ATGACGGAAAAAGAGATCGTGGATTACGTCGCCAGTCAAGTAACAACCGCGAAAAA
GTTGCGCGGAGGAGTTGTGTTTGTGGACGAAGTACCGAAAGGTCTTACCGGAAAAC
TCGACGCAAGAAAAATCAGAGAGATCCTCATAAAGGCCAAGAAGGGCGGAAAGATC
GCCGTGGTGTACAAGGGAGCAACCAACTTTTCCCTGCTGAAGCAGGCAGGCGACGT
GGAGGAGAATCCTGGACCCATGGTGAGCAAGGGCGAGGAGCTGTTCACCGGGGTGG
TGCCCATCCTGGTCGAGCTGGACGGCGACGTAAACGGCCACAAGTTCAGCGTGTCC
GGCGAGGGCGAGGGCGATGCCACCTACGGCAAGCTGACCCTGAAGTTCATCTGCAC
CACCGGCAAGCTGCCCGTGCCCTGGCCCACCCTCGTGACCACCCTGACCTACGGCG
TGCAGTGCTTCAGCCGCTACCCCGACCACATGAAGCAGCACGACTTCTTCAAGTCC
GCCATGCCCGAAGGCTACGTCCAGGAGCGCACCATCTTCTTCAAGGACGACGGCAA
CTACAAGACCCGCGCCGAGGTGAAGTTCGAGGGCGACACCCTGGTGAACCGCATCG
AGCTGAAGGGCATCGACTTCAAGGAGGACGGCAACATCCTGGGGCACAAGCTGGAG
TACAACTACAACAGCCACAACGTCTATATCATGGCCGACAAGCAGAAGAACGGCAT
CAAGGTGAACTTCAAGATCCGCCACAACATCGAGGACGGCAGCGTGCAGCTCGCCG
ACCACTACCAGCAGAACACCCCCATCGGCGACGGCCCCGTGCTGCTGCCCGACAAC
CACTACCTGAGCACCCAGTCCGCCCTGAGCAAAGACCCCAACGAGAAGCGCGATCA
CATGGTCCTGCTGGAGTTCGTGACCGCCGCCGGGATCACTCTCGGCATGGACGAGC
TGTACAAGTAATCTAGACAGACATGATAAGATACATTGATGAGTTTGGACAAACCA
CAACTAGAATGCAGTGAAAAAAATGCTTTATTTGTGAAATTTGTGATGCTATTGCT
TTATTTGTAACCATTATAAGCTGCAATAAACAAGTTAACAACAACAATTGCATTCA
TTTTATGTTTCAGGTTCAGGGGGAGGTGTGGGAGGTTTTTTAAAGCAAGTAAAACC
TCTACAAATGTGGTATGGGAGCTCGCGGCCGCAGGAACCCCTAGTGATGGAGTTGG
CCACTCCCTCTCTGCGCGCTCGCTCGCTCACTGAGGCCGGGCGACCAAAGGTCGCC
CGACGCCCGGGCTTTGCCCGGGCGGCCTCAGTGAGCGAGCGAGCGCGCAGCTGCCT
GCAGG

4. SP-hFc-ΔhFGF21

ATGAAGATCATCCTGTGGCTGTGTGTGTTCGGCCTGTTCCTGGCCACCCTGTTCCC
CATCAGCTGGCAGATGCCCGTGGAGTCCGGCCTGTCCTCCGAGGACTCCGCCAGCT
CCGAGAGCTTCGCCAAGCGCATCAAGCGCCACGGCATATCGGCCATGGTTAGATCT
GACAAAACTCACACATGCCCACCGTGCCCAGCACCTGAACTCCTGGGGGGACCGTC
AGTCTTCCTCTTCCCCCCAAAACCCAAGGACACCCTCATGATCTCCCGGACCCCTG
AGGTCACATGCGTGGTGGTGGACGTGAGCCACGAAGACCCTGAGGTCAAGTTCAAC
TGGTACGTGGACGGCGTGGAGGTGCATAATGCCAAGACAAAGCCGCGGGAGGAGCA

```
GTACAACAGCACGTACCGTGTGGTCAGCGTCCTCACCGTCCTGCACCAGGACTGGC
TGAATGGCAAGGAGTACAAGTGCAAGGTCTCCAACAAAGCCCTCCCAGCCCCCATC
GAGAAAACCATCTCCAAAGCCAAAGGGCAGCCCCGAGAACCACAGGTGTACACCCT
GCCCCCATCCCGGGAGGAGATGACCAAGAACCAGGTCAGCCTGACCTGCCTGGTCA
AAGGCTTCTATCCCAGCGACATCGCCGTGGAGTGGGAGAGCAATGGGCAGCCGGAG
AACAACTACAAGACCACGCCTCCCGTGCTGGACTCCGACGGCTCCTTCTTCCTCTA
CAGCAAGCTCACCGTGGACAAGAGCAGGTGGCAGCAGGGGAACGTCTTCTCATGCT
CCGTGATGCACGAGGCTCTGCACAACCACTACACGCAGAAGAGCCTCTCCCTGTCT
CCGGGTAAAGCCAGCGGAAGTGGCGGAGGAGGCGGTCACCCCATCCCTGACTCCAG
TCCTCTCCTGCAATTCGGGGGCCAAGTCCGGCAGCGGTACCTCTACACAGATGATG
CCCAGCAGACAGAAGCCCACCTGGAGATCAGGGAGGATGGGACGGTGGGGGGCGCT
GCTGACCAGAGCCCCGAAAGTCTCCTGCAGCTGAAAGCCTTGAAGCCGGGAGTTAT
TCAAATCTTGGGAGTCAAGACATCCAGGTTCCTGTGCCAGCGGCCAGATGGGGCCC
TGTATGGATCGCTCCACTTTGACCCTGAGGCCTGCAGCTTCCGGGAGAGGCTTCTT
GAGGACGGATACAATGTTTACCAGTCCGAAGCCCACGGCCTCCCGCTGCACCTGCC
AGGGAACAAGTCCCCACACCGGGACCCTGCACCCCGAGGACCAGCTCGCTTCCTGC
CACTACCAGGCCTGCCCCCCGCACTCCCGGAGCCACCCGGAATCCTGGCCCCCCAG
CCCCCCGATGTGGGCTCCTCGGACCCTCTGAGCATGGTGGGAGGGTCCCAGGGCCG
AAGCCCCAGCTACGAGTCCTAA
```

5. SP-ΔmIL-4

```
ATGGGTCTCAACCCCCAGCTAGTTGTCATCCTGCTCTTCTTTCTCGAATGTACCAG
GAGCCATATCCACGGATGCGACAAAAATCACTTGAGAGAGATCATCGGCATTTTGA
ACGAGGTCACAGGAGAAGGGACGCCATGCACGGAGATGGATGTGCCAAACGTCCTC
ACAGCAACGAAGAACACCACAGAGAGTGAGCTCGTCTGTAGGGCTTCCAAGGTGCT
TCGCATATTTTATTTAAAACATGGGAAAACTCCATGCTTGAAGAAGAACTCTAGTG
TTCTCATGGAGCTGCAGAGACTCTTTCGGGCTTTTCGATGCCTGGATTCATCGATA
AGCTGCACCATGAATGAGTCCAAGTCCACATCACTGAAAGACTTCCTGGAAAGCCT
AAAGAGCATCATGGACATGGATGACTCGTAG
```

6. O_{stat6}-P_{min}

```
AATAAAATATCTTTATTTTCATTACATCTGTGTGTTGGTTTTTTGTGTGAATCGAT
AGTACTAACATACGCTCTCCATCAAAACAAAACGAAACAAAACAAACTAGCAAAAT
AGGCTGTCCCCAGTGCAAGTGCAGGTGCCAGAACATTTCTCTGGCCTAACTGGCCG
GTACCCGCTGTTGCTCAATCGACTTCCCAAGAACAGCGCTGTTGCTCAATCGACTT
CCCAAGAACAGCAGGGATCTGGCCTCGGCGGCCAAGCTCCGCTGTTGCTCAATCGA
CTTCCCAAGAACAGCGCTGTTGCTCAATCGACTTCCCAAGAACAGCAGGGATCTGG
CCTCGGCGGCCAAGCTTAGACACTAGAGGGTATATAATGGAAGCTCGACTTCCAGC
TTGGCAATCCGGTACTGTTGGTAAA
```

Supplementary References

1. Kemmer, C. et al. A designer network coordinating bovine artificial insemination by ovulation-triggered release of implanted sperms. *J Control Release* **150**, 23-29 (2011).
2. Shirasu, M., Yoshikawa, K., Takai, Y., Nakashima, A., Takeuchi, H., Sakano, H., Touhara, K. Olfactory receptor and neural pathway responsible for highly selective sensing of musk odors. *Neuron* **81**, 165-178 (2014).
3. Fussenegger, M. et al. Streptogramin-based gene regulation systems for mammalian cells. *Nat. Biotechnol.* **18**, 1203-1208 (2000).
4. Dahoun, T., Grasso, L., Vogel, H. & Pick, H. Recombinant expression and functional characterization of mouse olfactory receptor mOR256-17 in mammalian cells. *Biochemistry* **50**, 7228-7235 (2011).
5. Keeley, M. B., Busch, J., Singh, R. & Abel, T. TetR hybrid transcription factors report cell signaling and are inhibited by doxycycline. *Biotechniques.* **39**, 529-536 (2005).
6. Bai, P. et al. A fully human transgene switch to regulate therapeutic protein production by cooling sensation. *Nat. Med.* **25**, 1266-1273 (2019).
7. Liu,Y. et al. Immunomimetic designer cells protect mice from methicillin-resistant Staphylococcus aureus infection. *Cell* **174**, 259-270 (2018).
8. Xie, M. et al. beta-cell-mimetic designer cells provide closed-loop glycemic control. *Science* **354**, 1296-1301 (2016).
