# Supplementary material for: AAV-delivered muscone-induced transgene system for treating chronic diseases in mice via inhalation
Source: Nat Commun. 2024 Feb 6;15:1122. doi: 10.1038/s41467-024-45383-z (PMC10847102; doi:10.1038/s41467-024-45383-z)
Supplement: Supplementary file 2 — Reporting Summary [file 41467_2024_45383_MOESM2_ESM.pdf]

nature portfolio

Corresponding author(s): Haifeng Ye

Last updated by author(s): Jan 2, 2024

Reporting Summary

Nature Portfolio wishes to improve the reproducibility of the work that we publish. This form provides structure for consistency and transparency in reporting. For further information on Nature Portfolio policies, see our Editorial Policies and the Editorial Policy Checklist.

Statistics

For all statistical analyses, confirm that the following items are present in the figure legend, table legend, main text, or Methods section.

n/a	Confirmed	
☐	☒	The exact sample size (*n*) for each experimental group/condition, given as a discrete number and unit of measurement
☐	☒	A statement on whether measurements were taken from distinct samples or whether the same sample was measured repeatedly
☐	☒	The statistical test(s) used AND whether they are one- or two-sided<br>*Only common tests should be described solely by name; describe more complex techniques in the Methods section.*
☒	☐	A description of all covariates tested
☒	☐	A description of any assumptions or corrections, such as tests of normality and adjustment for multiple comparisons
☐	☒	A full description of the statistical parameters including central tendency (e.g. means) or other basic estimates (e.g. regression coefficient) AND variation (e.g. standard deviation) or associated estimates of uncertainty (e.g. confidence intervals)
☐	☒	For null hypothesis testing, the test statistic (e.g. *F*, *t*, *r*) with confidence intervals, effect sizes, degrees of freedom and *P* value noted<br>*Give P values as exact values whenever suitable.*
☒	☐	For Bayesian analysis, information on the choice of priors and Markov chain Monte Carlo settings
☒	☐	For hierarchical and complex designs, identification of the appropriate level for tests and full reporting of outcomes
☒	☐	Estimates of effect sizes (e.g. Cohen's *d*, Pearson's *r*), indicating how they were calculated

Our web collection on statistics for biologists contains articles on many of the points above.

Software and code

Policy information about availability of computer code

Data collection	The expression levels of SEAP, luciferase, ΔhFGF21 and ΔmIL-4 in cells were quantified using a Synergy H1 hybrid multi-mode microplate reader with Gen5 software (version: 2.04).<br>The levels of cytokines (IL-4, IL-5, IL-13), IgE in mouse BLAF, and the metabolic indexes (TG, T-CHO, AST, ALT) in mouse plasma were quantified using a Synergy H1 hybrid multi-mode microplate reader with Gen5 software (version: 2.04).<br>Bioluminescence images of the mice were obtained using IVIS Lumina II in vivo imaging system (Perkin Elmer, USA).<br>Eosinophils and T lymphocytes in mouse BALF and lung were quantified using the LSRFortessa Flow Cytometer (BD Biosciences, BD FACSDiva™ software v9.0).<br>Indirect calorimetry tests of the mice were performed in metabolic cages of a Comprehensive Lab Animal Monitoring System (CLAMS, Columbus Instruments, USA).<br>Body compositions were measured at indicated time using AccuFat MRI system (AccuFat-1050, MAG-MED).<br>Liver and lung sections were observed using an upright microscope (BX53, Olympus) equipped with an Olympus digital camera.<br>Whole blood was analyzed for complete blood count using the Sysmex XT-2000iV hematology analyzer (Sysmex).<br>Flow cytometry data were collected using the BD LSRFortessaTM Flow Cytometer (BD Biosciences) equipped with BD FACS Diva™ Software (version: 9.0) .<br>The quantitative analysis of muscone was performed on an Agilent 7890B gas chromatography system coupled with an Agilent 7000D triple quadrupole mass spectrometer (Agilent Technologies, USA).<br>RNA sequencing was performed via Illumina platform (Illumina Novaseq 6000, CA).
Data analysis	We used GraphPad (version 8) to perform statistical analysis.<br>We used Gen5 software (version 2.04) to analyze absorbances of different samples.

Bioluminescence values of the mice were analyzed using Living Image® software (version 4.3.1) .
We used FlowJo software (version 9.0) to analyze flow cytometry data.
The analyses of RNAseq data were performed by R Software (version 4.0.2).
The Volume of O2 consumption and CO2 expiration were analyzed using Oxymax® software (version 5.51).
The fat mass and lean mass were analyzed using AccuFat 1050.
The images of liver sections were obtained by cellSens Standard software (version 1.5).
Gene set enrichment analysis (GSEA) was performed on http://www.broadinstitute.org/gsea/index.jsp.

For manuscripts utilizing custom algorithms or software that are central to the research but not yet described in published literature, software must be made available to editors and reviewers. We strongly encourage code deposition in a community repository (e.g. GitHub). See the Nature Portfolio guidelines for submitting code & software for further information.

Data

Policy information about availability of data

All manuscripts must include a data availability statement. This statement should provide the following information, where applicable:
- Accession codes, unique identifiers, or web links for publicly available datasets
- A description of any restrictions on data availability
- For clinical datasets or third party data, please ensure that the statement adheres to our policy

All data associated with this study are present in the paper or the Supplementary Information. The raw data of RNA-seq was uploaded into the NCBI Sequencing Read Archive (SRA) under accession number PRJNA1011482 [https://www.ncbi.nlm.nih.gov/search/all/?term=PRJNA1011482] and the BioProject access link is https://dataview.ncbi.nlm.nih.gov/object/PRJNA1011482?reviewer=40tpnfqk3enotdg3b3vpvmom3b. The NCBI SRA accession number (PRJNA1011482) is currently available and is publicly released. The remaining data are available within the Article, Supplementary Information or Source Data file. Source data are provided with this paper. All genetic components related to this paper are available with a material transfer agreement and can be requested from H.Y. (hfye@bio.ecnu.edu.cn).

Research involving human participants, their data, or biological material

Policy information about studies with human participants or human data. See also policy information about sex, gender (identity/presentation), and sexual orientation and race, ethnicity and racism.

Reporting on sex and gender	No human subjects were used
Reporting on race, ethnicity, or other socially relevant groupings	None.
Population characteristics	None.
Recruitment	None.
Ethics oversight	None.

Note that full information on the approval of the study protocol must also be provided in the manuscript.

Field-specific reporting

Please select the one below that is the best fit for your research. If you are not sure, read the appropriate sections before making your selection.

[x] Life sciences [ ] Behavioural & social sciences [ ] Ecological, evolutionary & environmental sciences

For a reference copy of the document with all sections, see nature.com/documents/nr-reporting-summary-flat.pdf

Life sciences study design

All studies must disclose on these points even when the disclosure is negative.

Sample size	No statistical methods were used to predetermine sample size. For vitro study, at least three biologically independent experimental replicates were performed for statistical analyses, according to standard scientific conventions. For in vivo study, 4-5 mice per group were determined by the reproducibility of bioluminescence images. Sample size was determined based on the previous published studies with n=3-5 mice per group, yielding statistically significant difference (Vincent RL., et al., Science, 2023; Wu Y., et al., Nat Biomed Eng, 2021; Cho JH., et al., Cell, 2018; Allen GM., et al., Science, 2022). The sample size used for each experiment is indicated at the corresponding figure legend in the manuscript.
Data exclusions	No data were excluded.
Replication	The number of independent experiments is specified in each figure legend, with at least 3 independent experiments, unless otherwise specified.

Randomization	Physical randomization. All the animals used were randomly selected from the delivered pool. Mice 6-8 weeks old were randomly selected from cages, divided into groups for the studies.
Blinding	For all the animal and cell experiments, the investigators were blinded to the group allocations during data analysis.

Reporting for specific materials, systems and methods

We require information from authors about some types of materials, experimental systems and methods used in many studies. Here, indicate whether each material, system or method listed is relevant to your study. If you are not sure if a list item applies to your research, read the appropriate section before selecting a response.

Materials & experimental systems

n/a	Involved in the study	
☐	☒	Antibodies
☐	☒	Eukaryotic cell lines
☒	☐	Palaeontology and archaeology
☐	☒	Animals and other organisms
☒	☐	Clinical data
☒	☐	Dual use research of concern
☒	☐	Plants

Methods

n/a	Involved in the study	
☒	☐	ChIP-seq
☐	☒	Flow cytometry
☒	☐	MRI-based neuroimaging

Antibodies

Antibodies used	BV510 Live/Dead (catalog no. 423101, Biolegend, USA)<br>Alexa Fluor®-700 anti-mouse CD45 (catalog no. 103127, clone 30-F11, Biolegend, USA)<br>KIRAVIA Blue 520 anti-mouse F4/80 (catalog no. 123161, clone BM8, Biolegend, USA)<br>Brilliant Violet 421 anti-mouse CD11c (catalog no. 117329, clone N418, Biolegend, USA)<br>APC/Cyanine7-anti-mouse CD11b (catalog no. 101226, clone M1/70, Biolegend, USA)<br>PE anti-mouse CD170 (Siglec-F) (catalog no. 155505, clone S17007L, Biolegend, USA)<br>FITC anti-mouse CD3ε (catalog no.100306, clone 145-2C11, Biolegend, USA)<br>All antibodies were purchased from Biolegend and were diluted at 1:200.
Validation	All antibodies sourced from commercial corporation are well-validated by the manufacturer and are widely used in the scientific community. The information of antibodies used in this study are available through the manufacturers' websites.<br>BV510 Live/Dead (423101, Biolegend)-PMID: 24277150, 24973457, 25008920, 28287113, 33261178, 33046889, 32434881, 33459871, 29900048, 28428881. [https://www.biolegend.com/en-gb/products/zombie-aqua-fixable-viability-kit-8444]<br>Alexa Fluor®-700 anti-mouse CD45 (103127, Biolegend)-PMID:22343916, 22547694, 31686028, 33730589, 34125490, 31704965, 31996771, 29628141. [https://www.biolegend.com/en-gb/products/alexa-fluor-700-anti-mouse-cd45-antibody-3407]<br>KIRAVIA Blue 520 anti-mouse F4/80 (123161, Biolegend)-PMID:18372338, 19509298, 23554311, 24711449, 24657529. [https://www.biolegend.com/en-gb/products/kiravia-blue-520-anti-mouse-f480-antibody-19134]<br>Brilliant Violet 421 anti-mouse CD11c (117329, Biolegend)-PMID:16985170, 17513751, 19494255, 16818746, 18252869, 30824325, 32515732. [https://www.biolegend.com/en-gb/products/brilliant-violet-421-anti-mouse-cd11c-antibody-7149]<br>APC/Cyanine7-anti-mouse CD11b (101226, Biolegend)-PMID: 22814747, 23144497, 25101794, 33440159, 36110849, 16493044, 18390836. [https://www.biolegend.com/en-gb/products/apc-cyanine7-anti-mouse-human-cd11b-antibody-3930]<br>PE anti-mouse CD170 (Siglec-F) (155505, Biolegend)-PMID: 33662276, 34727095, 34115982, 36110849, 32284604, 34193600. [https://www.biolegend.com/en-gb/products/pe-anti-mouse-cd170-siglec-f-antibody-16372]<br>FITC anti-mouse CD3ε (100306, Biolegend)-PMID: 16177080, 18390836, 17641043, 29273790, 31341173, 18796632. [https://www.biolegend.com/en-gb/products/fitc-anti-mouse-cd3epsilon-antibody-23]

Eukaryotic cell lines

Policy information about cell lines and Sex and Gender in Research

Cell line source(s)	Hana3A cells were kindly provided by Professor Dr. Martin Fussenegger, Department of Biosystems Science and Engineering, ETH Zürich. The HEK-293T (CRL-11268, ATCC), HeLa (CCL-2, ATCC) and human mesenchymal stem cells (hMSC-TERT; SCRC-4000, ATCC) were obtained from ATCC and cultured in DMEM supplemented with 10% FBS.
Authentication	None of the cell lines are authenticated.
Mycoplasma contamination	No mycoplasma contamination was detected for all cells.
Commonly misidentified lines<br>(See ICLAC register)	No misidentified cell lines have been used in this study.

Animals and other research organisms

Policy information about studies involving animals; ARRIVE guidelines recommended for reporting animal research, and Sex and Gender in Research

Laboratory animals	Adult female BALB/c mice (8-week-old) and male C57BL/6 mice (8-week-old) were obtained from the ECNU (East China Normal University) Laboratory Animal Center. The mice were kept in an animal house maintained at 22 ± 2°C, with a 12-hour light-dark cycle and free access to food and water.
Wild animals	None.
Reporting on sex	The sex of mice was taken into account in the study design. For the NAFLD model, male C57BL/6 mice are commonly chosen to construct the NAFLD model due to their susceptibility to diet-induced obesity and metabolic complications (Changyu Zhu et al., Sci Transl Med., 2018). For allergic asthma model, female BALB/c mice are selected to construct the allergic asthma model because female mice have been reported to be developed a more pronounced type of allergic airway inflammation than male mice after OVA challenge (Takeda et al., Respirology, 2013; Weiss et al., Immunohorizons, 2021).
Field-collected samples	None.
Ethics oversight	The protocol involved in this study was approved by the East China Normal University (ECNU) Animal Care and Use Committee (protocol ID: m20200213 and m20210114).

Note that full information on the approval of the study protocol must also be provided in the manuscript.

Flow Cytometry

Plots

Confirm that:

- [x] The axis labels state the marker and fluorochrome used (e.g. CD4-FITC).
- [x] The axis scales are clearly visible. Include numbers along axes only for bottom left plot of group (a 'group' is an analysis of identical markers).
- [x] All plots are contour plots with outliers or pseudocolor plots.
- [x] A numerical value for number of cells or percentage (with statistics) is provided.

Methodology

Sample preparation	Collected cells were washed with cell staining buffer for 3 times, and then stained with specific antibodies. At least 5000 events were analyzed per sample.
Instrument	BD LSRFortessaTM Flow Cytometer (BD Biosciences)
Software	Data collection: BD FACS Diva™ Software (version: 9.0) .<br>Data analysis: FlowJo 10.8.1
Cell population abundance	No sorting was conducted and single lymphocyte suspensions of spleens and peripheral blood were analyzed.
Gating strategy	Generally, cells wee first gated on FSC/SSC. Surface-antigen gating was performed on the live cell population.

- [x] Tick this box to confirm that a figure exemplifying the gating strategy is provided in the Supplementary Information.
